# SUMOylation of ROR-γt inhibits IL-17 expression and inflammation via HDAC2

**DOI:** 10.1038/s41467-018-06924-5

**Published:** 2018-10-30

**Authors:** Amir Kumar Singh, Prashant Khare, Abeer Obaid, Kevin P. Conlon, Venkatesha Basrur, Ronald A. DePinho, K. Venuprasad

**Affiliations:** 1 Baylor Institute for Immunology Research, Baylor Scott & White Research Institute, Dallas, TX 75204 USA; 2 Department of Pathology, University of Michigan, Ann Arbor, MI 48109 USA; 3 Department of Cancer Biology, The University of Texas MD Anderson Cancer Center, Houston, TX 77030 USA

## Abstract

Dysregulated ROR-γt-mediated IL-17 transcription is central to the pathogenesis of several inflammatory disorders, yet the molecular mechanisms that govern the transcription factor activity of ROR-γt in the regulation of IL-17 are not fully defined. Here we show that SUMO-conjugating enzyme Ubc9 interacts with a conserved GKAE motif in ROR-γt to induce SUMOylation of ROR-γt and suppress IL-17 expression. Th17 cells expressing SUMOylation-defective ROR-γt are highly colitogenic upon transfer to Rag1^–/–^ mice. Mechanistically, SUMOylation of ROR-γt facilitates the binding of HDAC2 to the IL-17 promoter and represses IL-17 transcription. Mice with conditional deletion of HDAC2 in CD4^+^ T cells have elevated IL-17 expression and severe colitis. The identification of the Ubc9/ROR-γt/HDAC2 axis that governs IL-17 expression may open new venues for the development of therapeutic measures for inflammatory disorders.

## Abstract (web summary)

Interleukin-17 (IL-17)-secreting CD4 T cells (Th17) are induced by the master transcription factor RORγt, and are important for anti-fungal immunity and inflammatory responses. Here the authors show that Ubc9-mediated SUMOylation of RORγt induces HDAC2 binding to IL-17 promoter for suppressing IL-17 production in Th17 cells.

## Introduction

While inflammation is protective against microbial infections and tissue injury, uncontrolled inflammation can cause host tissue damage that may lead to autoimmunity and malignancy. Emerging evidence points to a critical role for interleukin-17 (IL-17) in both host defense and inflammation^1,2^. IL-17 is produced by a variety of immune cells, including the T_H_17 subset of helper T cells, γδ T cells, and innate lymphoid cells^1,2^. IL-17 triggers inflammation by inducing multiple cytokines and chemokines, which in turn recruit neutrophils and macrophages that contribute to tissue damage^3^. While transient IL-17 expression in response to infection is protective, dysregulated IL-17 expression is thought to be foundational to the pathogenesis of several human inflammatory diseases including psoriasis, ankylosing spondylitis, rheumatoid arthritis, multiple sclerosis, and inflammatory bowel diseases^4^.

The orphan nuclear receptor ROR-γt is the key transcription factor that induces IL-17 expression^5,6^. Structurally, ROR-γt consists of a ligand-independent activation function 1 helix, a DNA-binding domain, a flexible hinge domain, and a C-terminal ligand-binding domain^7^. The two zinc-finger motifs within the DNA-binding domain recognize the ROR response elements within the IL-17 promoter to induce IL-17 expression^7^. Accordingly, ROR-γt regulation has emerged as an area of active study for potential pharmacological interventions^8^. However, a clear understanding of ROR-γt regulation is currently lacking, yet is absolutely necessary to target ROR-γt effectively.

Post-translational modification by small (~12 kDa) ubiquitin-like modifier (SUMO) proteins involves covalent attachment of a SUMO to a lysine residue in the target protein^9–11^. Like ubiquitination, SUMO conjugation involves a cascade of biochemical reactions that involves E1, E2, and E3 enzymes. Ubc9 is the only E2 enzyme that is used by the SUMO pathway as a conjugation enzyme to transfer SUMO to the substrate proteins^9–11^. By influencing stability, intracellular localization, interaction with partners, and activity of target proteins, SUMOylation affects several biological processes including the cell cycle, DNA repair, chromatin dynamics, gene transcription, and inflammation^9–11^.

In this study, we show that Ubc9 interacts with and targets ROR-γt for SUMOylation and inhibits IL-17 expression. We demonstrate that the T cells expressing SUMOylation-defective ROR-γt are highly colitogenic upon transfer to Rag1^–/–^ mice. Mechanistically, SUMOylation of ROR-γt facilitates the binding of HDAC2 to the IL-17 promoter and represses IL-17 transcription. Thus, we uncover a mechanism by which IL-17 expression is regulated, which could be exploited therapeutically in inflammatory diseases.

## Results

### ROR-γt associates with Ubc9

**Fig. 1:**
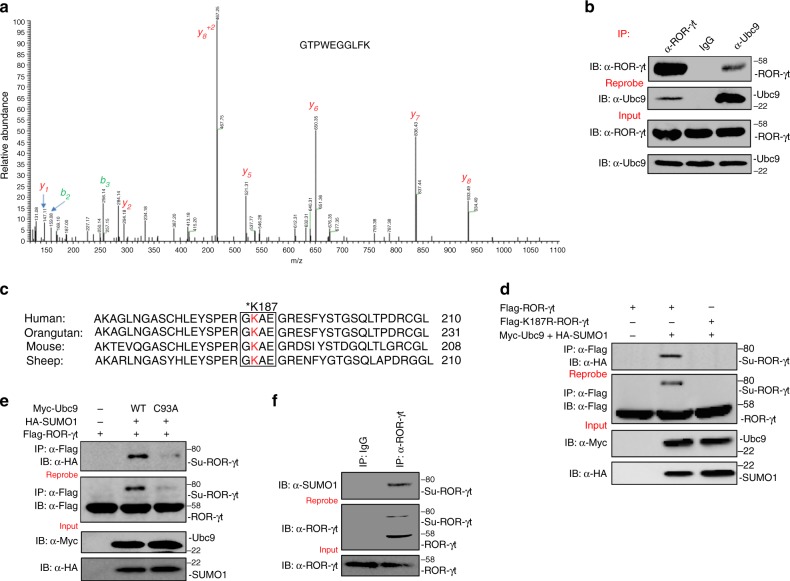
Ubc9 interacts with and SUMOylates ROR-γt. **a** Lysate was prepared from cLPLs of WT mice and subjected to immunoprecipitation with anti–ROR-γt antibody or control IgG antibody. The precipitated proteins were subjected to SDS-PAGE and in-gel digestion. The resulting peptides were analyzed by high-resolution MS/MS. Ubc9 (SwissProt #P63280) was identified as a specific interactor of ROR-γt protein. An MS/MS spectrum of the peptide ^50^GTPWEGGLFK^59^ ([M + H]^+2^ = 546.27 *m/z*) belonging to Ubc9 is shown. Observed *b*- and *y-*ions are indicated. **b** Lysates from cLPLs of WT mice were immunoprecipitated with anti-ROR-γt, anti-Ubc9 antibody, or control IgG antibody, and immunoblot analysis with antibody against ROR-γt or Ubc9 was performed. **c** Sequence alignment was conducted for ROR-γt. The box indicates the conserved SUMOylation (Ψ-K-X-D/E) motif. **d** Total cell lysates from 293 T cells transfected with Myc-Ubc9, HA-SUMO1, and either Flag-ROR-γt or Flag-K187R-ROR-γt were immunoprecipitated with anti-Flag antibody. The immunoprecipitates were analyzed by immunoblot assay with anti-HA antibody to detect the SUMOylated form of ROR-γt. **e** Total cell lysates from 293 T cells transfected with Flag-ROR-γt, HA-SUMO1, and either Myc-Ubc9 or Myc-Ubc9-C93A were immunoprecipitated with anti-Flag antibody and immunoblotted with anti-HA antibody to detect the SUMOylated form of ROR-γt. **f** Lysates were prepared from cLPLs of WT mice and immunoprecipitated with anti–ROR-γt antibody or control IgG antibody. The immunoprecipitates were immunoblotted with anti-SUMO1 antibody. The data are representative of three or more independent experiments

Our previous work established that the ubiquitin ligase Itch targets ROR-γt for ubiquitination and promotes its degradation^12,13^. To further delineate the molecular mechanism by which ROR-γt function is regulated, we adopted a proteomics approach to identify additional components in the transcriptional complex. Given the central role of colonic lamina propria lymphocytes (cLPLs) in gut homeostasis and inflammation, we isolated cLPLs from C57BL/6 (WT) mice followed by lysis and ROR-γt immunoprecipitation using a validated anti–ROR-γt antibody. The precipitated proteins were resolved by SDS-PAGE and subjected to mass spectrometry (MS) analysis after in-gel digestion with trypsin. MS spectra corresponding to a specific Ubc9 peptide were present in anti–ROR-γt precipitates but not in control IgG precipitates (Fig. 1a). The MS findings were further validated in co-immunoprecipitation studies in 293 T cells transiently transfected with expression vectors encoding Flag-tagged ROR-γt (Flag-ROR-γt) and Myc-tagged Ubc9 (Myc-Ubc9). Immunoprecipitation with either anti-Flag or anti-c-Myc antibodies showed that anti-Flag immunoprecipitates contained Myc-Ubc9 and that anti-c-Myc pulled down Flag-ROR-γt (Supplementary Fig. 1a). Finally, endogenous ROR-γt-Ubc9 interactions in cLPLs lysates were confirmed in anti–ROR-γt and anti-Ubc9 co-immunoprecipitates (Fig. 1b). Together, these assays establish that ROR-γt physically interacts with Ubc9.

### Ubc9 recognizes the GKAE motif and SUMOylates ROR-γt

Since Ubc9 is a SUMO-E2 enzyme that facilitates SUMOylation^9–11^, we hypothesized that ROR-γt function may be regulated by its SUMOylation. In most cases, Ubc9 recognizes the consensus Ψ-K-X-E/D motif (where Ψ is a hydrophobic residue, K is the lysine conjugated to SUMO, X is any amino acid, and D or E is an acidic residue) on its protein substrates^9–11^. Interestingly, sequence alignment of ROR-γt revealed a highly conserved GKAE consensus motif (Fig. 1c).

As a first step in assessing whether ROR-γt is SUMOylated, the lysine residue of the consensus sequence was mutated to arginine (K187R-ROR-γt). 293 T cells were transfected with either Flag-ROR-γt or K187R-ROR-γt (also Flag tagged) along with Myc-Ubc9 and HA-SUMO1. Immunoprecipitation of ROR-γt (using anti-Flag antibody) followed by western blot analysis with anti-HA antibody revealed an upshifted band only in the cells expressing wild type (WT) ROR-γt and no band in the cells expressing K187R-ROR-γt (Fig. 1d). Together, these data are consistent with the possibility that Ubc9/SUMO1 SUMOylates ROR-γt at K187 within the GKAE motif. To confirm that the upshifted band is SUMOylated ROR-γt, we washed and then reprobed the same membrane with anti-Flag antibody. This revealed that WT-ROR-γt but not K187R-ROR-γt is SUMOylated (Fig. 1d). To further confirm the SUMOylation of ROR-γt, we used a catalytically inactive Ubc9 mutant in which a conserved cysteine is replaced with an alanine (Ubc9-C93A)^14^. Expression of the Ubc9-C93A mutant with ROR-γt did not result in its SUMOylation, indicating that the catalytic activity of Ubc9 is essential for the SUMOylation of ROR-γt (Fig. 1e). Further analysis of primary cLPLs and in vitro differentiated Th17 cells confirmed the presence of the SUMOylated form of ROR-γt (Fig. 1f and Supplementary Fig. 1b). Densitometry analysis showed that about 21%, 17%, and 11% of ROR-γt was SUMOylated in 293 T cells, cLPLs, and in vitro differentiated Th17 cells, respectively (Supplementary Fig. 1c–e). In addition, SUMO2 and SUMO3, the two other isoforms of SUMO, could promote SUMOylation of ROR-γt. We found that SUMO2 and SUMO3 were much less efficient in SUMOylating ROR-γt compared to SUMO1 (Supplementary Fig. 1f).

### SUMOylation of ROR-γt inhibits IL-17 expression

**Fig. 2:**
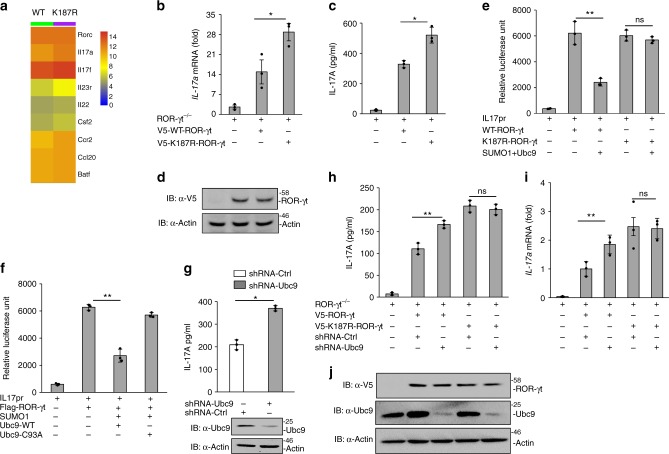
SUMOylation of ROR-γt inhibits IL-17 expression. **a** Data show heatmap visualization of upregulated genes determined by RNA seq analysis in ROR-γt^*–/–*^ CD4^+^ T cells transduced with either WT ROR-γt or K187R-ROR-γt and differentiated under Th17-inducing conditions. **b** Real-time PCR analysis was conducted for *IL-17a* mRNA expression in ROR-γt^*–/–*^ CD4^+^ T cells transduced with either WT ROR-γt or K187R-ROR-γt. The relative fold change in the mRNA levels of the genes was normalized against ROR-γt^–/–^ cells as compared with lentivirus-transduced ROR-γt^–/–^ cells. **c** ELISA was conducted for IL-17A secretion in the culture supernatant of ROR-γt^–/–^ cells transduced with lentivirus encoding V5-tagged WT-ROR-γt or K187R-ROR-γt. **d** Immunoblot analysis of the expression of lentiviral constructs of V5-ROR-γt in ROR-γt^–/–^ cells. **e** Luciferase assay was conducted of lysates from Jurkat T cells transfected with various combinations (below plot) of the IL-17-promoter-driven luciferase plasmid (pGL4-mIL17p) and plasmid-encoded HA-SUMO1, Myc-Ubc9, Flag-ROR-γt, and/or Flag-K187R-ROR-γt. **f** Luciferase assay was conducted of lysates from Jurkat T cells transfected with various combinations (below plot) of plasmid pGL4-mIL17p along with Flag-ROR-γt, HA-SUMO1, and either Myc-Ubc9 or Myc-Ubc9-C93A. Results are presented in relative luciferase units (RLU). **g** Data show Ubc9 knockdown by Ubc9-specific shRNA (shUbc9) or control shRNA (shCtrl) in WT cLPLs that were stimulated with PMA and ionomycin. ELISA was performed to measure IL-17A secretion in culture supernatant of Ubc9 knockdown cLPLs as well as immunoblotting showing knockdown of Ubc9. **h** ELISA was performed to measure the IL-17A secretion in the culture supernatant of Ubc9 knockdown cells expressing either WT-ROR-γt or K187R-ROR-γt. **i** Real-time PCR analysis for *IL-17a* mRNA expression, in which the relative fold change in the mRNA levels of the genes has been normalized against ROR-γt-expressing cells treated with control shRNA compared to Ubc9-specific shRNA. **j** Immunoblot analysis shows Ubc9 knockdown in CD4^+^ T cells expressing either WT-ROR-γt or K187R-ROR-γt. Data are from one experiment representative of three independent experiments with similar results. *p *<* 0.05, **p *<* 0.01, ns: non-significant (two-tail *t* test) error bars are S.D.

To gain insight into the functional consequence of ROR-γt SUMOylation, ROR-γt^−/−^ mouse-derived T cells were reconstituted with either WT-ROR-γt or the K187R-ROR-γt mutant (SUMOylation-defective ROR-γt)^12^. The cells were then differentiated under Th17-inducing conditions and whole-genome transcriptome analysis (RNA seq) of the RNA isolated from these cells was performed. As shown in Fig. 2a–c and Supplementary Fig. 2, transcriptome, real-time PCR, and ELISA analysis showed that several Th17-associated genes, including IL-17A, *IL-17f*, and *IL-23r*, were upregulated in Th17 cells expressing the SUMOylation-defective K187R-ROR-γt mutant form of ROR-γt. The expression of WT and mutant K187R-ROR-γt was analyzed by immunoblotting (Fig. 2d). To investigate if SUMOylation of ROR-γt at the K187 regulates IL-17 transcription, we performed IL-17-promoter-driven luciferase assay in Jurkat T cells that were transfected with WT-ROR-γt or K187R-ROR-γt along with Ubc9, SUMO1, and pGL4-mIL-17 promoter constructs. Following PMA and ionomycin stimulation, reporter analysis revealed that the WT-ROR-γt but not the K187R-ROR-γt mutant inhibited IL-17-promoter-driven luciferase activity, suggesting that SUMOylation of ROR-γt inhibits IL-17 expression (Fig. 2e). Furthermore, WT-Ubc9, but not the catalytic mutant Ubc9-C93A, inhibited IL-17-promoter-driven luciferase activity (Fig. 2f). Finally, shRNA-mediated depletion of Ubc9 in cLPLs revealed that Ubc9 depletion substantially elevated IL-17A expression, consistent with the model that SUMOylation of ROR-γt represses IL-17 expression (Fig. 2g).

To further confirm that Ubc9-mediated SUMOylation of ROR-γt inhibits IL-17 expression, we depleted Ubc9 using shRNA in ROR-γt^–/–^ CD4^+^ T cells expressing either WT-ROR-γt or K187R-ROR-γt. Ubc9 depletion in CD4^+^ T cells expressing WT-ROR-γt but, not K187R-ROR-γt, resulted in elevated IL-17 expression (Fig. 2h–j). Together, these findings reinforce the concept that SUMOylation of ROR-γt inhibits IL-17 expression.

### SUMOylation-defective Th17 cells are highly colitogenic

**Fig. 3:**
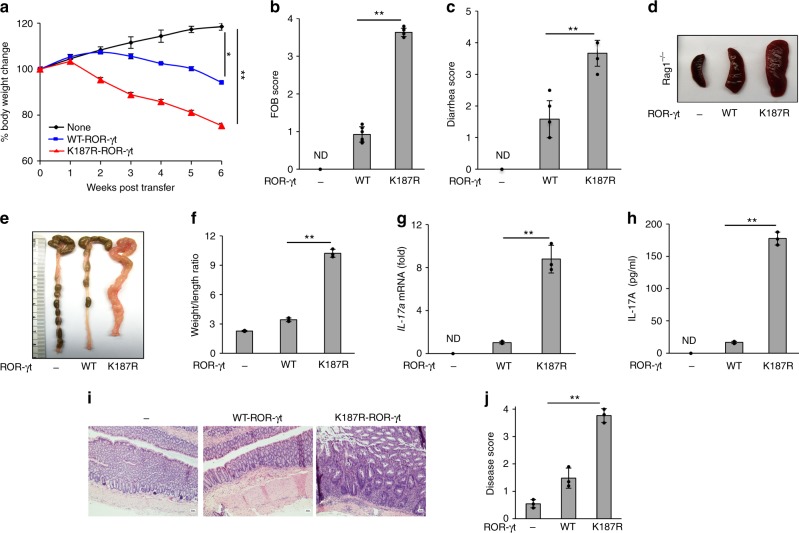
Th17 cells expressing SUMOylation-defective ROR-γt induce severe colitis in mice. ROR-γt^–/–^ CD4^+^ T cells transduced with lentivirus expressing WT-ROR-γt or a SUMOylation-deficient mutant ROR-γt were grown under Th17-polarizing conditions and adoptively transferred to Rag1^–/–^ mice. Data shows **a** body weight, **b** fecal occult blood score, **c** diarrhea score, **d** spleen size, and **e**, **f** colonic weight-to-length ratio for Rag1^–/–^mice (*n* *=* 4 per group) given an intraperitoneal injection of ROR-γt^–/–^ cells (None), Th17 cells expressing wild-type ROR-γt (WT-ROR-γt), or Th17 cells expressing SUMOylation-deficient mutant ROR-γt (K187R-ROR-γt) and monitored for 6 weeks. **g** Real-time PCR was used to analyze *IL-17a* mRNA. **h** ELISA was used to measure IL-17A secretion in the explant colon culture of Rag1^–/–^ mice as in **a**–**f** (*n* *=* 3). Results are presented relative to those of Rag1^–/–^ mice given ROR-γt^–/–^ cells. **i** Microscopy shows H&E-stained colonic sections. **j** Disease scores of those sections are provided from Rag1^–/–^ mice as in **a**–**f** (*n* *=* 3). ND: not detected. Data are from one experiment representative of three independent experiments with similar results. *p *<* 0.05, **p *<* 0.01 (two-tail *t* test) error bars are S.D.

To investigate the physiological impact of ROR-γt SUMOylation on the regulation of IL-17-mediated inflammation in vivo, we utilized the Th17 cell adoptive transfer colitis model^15,16^. Naive CD4^+^ T cells from ROR-γt^−/−^ mice were reconstituted with WT-ROR-γt or the K187R-ROR-γt mutant and these cells were differentiated under Th17-polarizing conditions. The live cells were recovered using Lympholyte, and 5 × 10^5^ cells were adoptively transferred into Rag1^–/–^ mice^12^. The recipient mice were monitored for signs of disease, including weight loss, fecal blood, and loose stool. The mice were killed 6 weeks after transfer, and the severity of colitis and IL-17 expression was assayed. Rag1^−/−^ mice that received Th17 cells expressing a SUMOylation-deficient mutant of ROR-γt (K187R-ROR-γt) exhibited greater loss of body weight, higher fecal occult blood and diarrhea scores, splenomegaly, and higher weight-to-length ratio of the colon compared with host mice that received Th17 cells expressing WT-ROR-γt (Fig. 3a–f). As expected, the expression of *IL-17a* mRNA was higher in the colonic mucosa, and the secretion of IL-17A was higher in the colonic explant culture of Rag1^−/−^ mice that received Th17 cells expressing K187R-ROR-γt (Fig. 3g, h). Histologic analysis documented greater infiltration of inflammatory cells, more crypt damage, and higher disease scores in the Rag1^−/−^ mice that received Th17 cells expressing K187R-ROR-γt relative to host mice that received Th17 cells expressing WT-ROR-γt (Fig. 3i, j). No gross signs of autoimmunity were observed in the liver, kidney, or spinal cord of these mice (Supplementary Fig. 3).

To further confirm the colitogenicity of SUMOylation-defective Th17 cells, adoptive transfer experiments were performed with undifferentiated ROR-γt^–/–^ CD4^+^ T cells expressing either WT-ROR-γt or K187R-ROR-γt into Rag1^–/–^ mice. Rag1^–/–^ mice that received T cells expressing K187R-ROR-γt exhibited greater loss of body weight, higher diarrhea scores, and higher weight-to-length ratio of the colon compared to mice that received T cells expressing WT-ROR-γt (Supplementary Fig. 4a-d). These mice exhibited higher *IL-17a* mRNA levels in the colonic mucosa, and colonic cultures showed higher IL-17A secretion than the WT-ROR-γt controls (Supplementary Fig. 4e-f); and, correspondingly, histologic analysis showed greater infiltration of inflammatory cells, more crypt damage, and higher diseases scores in the Rag1^–/–^ mice receiving T cells expressing K187R-ROR-γt relative to those receiving T cells expressing WT-ROR-γt (Supplementary Fig. 4g–h). Together, these findings support a critical role for SUMOylation of ROR-γt in the control of pathogenic colonic inflammation.

### SUMOylation facilitates the binding of HDAC2 to ROR-γt

Next, we sought to investigate how SUMOylation of ROR-γt represses IL-17 transcription. We recently reported that the ubiquitin ligase Itch targets ROR-γt for ubiquitination. Generally, the SUMO and ubiquitination pathways have an antagonistic relationship^9^. However, in some instances, SUMOylation has been shown to promote protein ubiquitination^17,18^. Therefore, we investigated whether SUMOylation and ubiquitination occur at the same lysine residue and thus co-regulate ROR-γt ubiquitination. To that end, we transiently transfected WT-ROR-γt and K187R-ROR-γt along with HA-Ub and Myc-Itch, which showed no change in ubiquitination of K187R-ROR-γt compared to WT-ROR-γt (Supplementary Fig. 5a). Similarly, the K187R-ROR-γt mutation did not affect ROR-γt protein turnover **(**Supplementary Fig. 5b). We conclude that SUMOylation regulates ROR-γt function independently of its ubiquitination.

Since the DNA binding ability of the transcription factors can be modulated by SUMOylation of transcription factors^9–11^, we analyzed if the K187R mutation of ROR-γt affected its binding to the IL-17 promoter. As shown in Supplementary Fig. 5c, chromatin immunoprecipitation (ChIP) analysis showed similar binding of K187R-ROR-γt and WT-ROR-γt. Similarly, the K187R-ROR-γt mutant interacted with Foxp3 and Runx1 similar to WT-ROR-γt, suggesting that SUMOylation does not affect the interaction of ROR-γt with Foxp3 and Runx1 (Supplementary Fig. 5d–e).

**Fig. 4:**
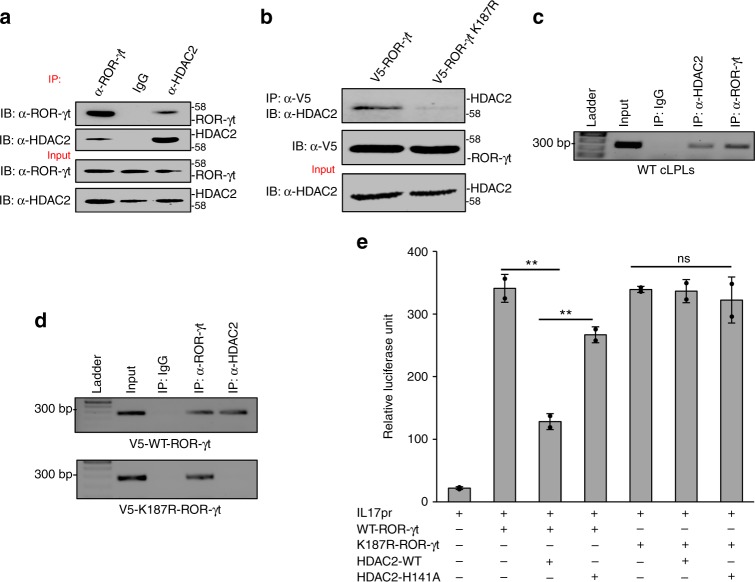
SUMOylation facilitates HDAC2-to-ROR-γt binding and inhibits IL-17 expression. **a** Lysates from cLPLs of WT mice were immunoprecipitated with anti-ROR-γt, anti-HDAC2 antibody, or control IgG antibody and immunoblotted with antibody against ROR-γt or HDAC2. **b** Transduced ROR-γt^–/–^ CD4^+^ T cells expressing either WT-ROR-γt or the K187R-ROR-γt were immunoprecipitated with anti-V5 antibody and immunoblot with antibody against V5 or HDAC2. **c** Binding of HDAC2 to the IL-17 promoter was assessed by ChIP analysis using a DNA-protein complex from cLPLs of WT mice that was immunoprecipitated with anti-ROR-γt, anti-HDAC2, or control IgG antibody. **d** SUMOylation of ROR-γt facilitates HDAC2 recruitment to the IL-17 promoter. ChIP analysis was conducted using DNA-protein complex from transduced ROR-γt^–/–^ CD4^+^ T cells expressing either WT-ROR-γt or the K187R-ROR-γt and immunoprecipitated with anti–ROR-γt, anti-HDAC2, or control IgG antibody. **e** A luciferase assay was performed of lysates from Jurkat T cells transfected with various combinations (below plot) of IL*-*17-promoter-driven luciferase plasmid (pGL4-mIL17p), plasmid encoding WT-ROR-γt or K187R-ROR-γt along with either HDAC2-WT or HDAC2-H141A. Results are presented in relative luciferase units (RLU). Data are from one experiment representative of three or more independent experiments with similar results. *p *<* 0.05 and **p *<* 0.01, ns: non-significant (two-tail *t* test) error bars are S.D.

It has been shown that SUMOylation represses the function of transcription factors by recruiting histone-modifying enzymes^9–11^. Intriguingly, our MS data identified HDAC2 as a potential ROR-γt-interacting protein (Supplementary Fig. 6a; peptide ^202^YGEYFPGTGDLR^213^). These data, coupled with the purported role of HDAC2-mediated histone deacetylation as a negative regulator of inflammation^19–22^, prompted us to hypothesize that HDAC2 binds to ROR-γt to inhibit IL-17 expression. To address this possibility, we immunoprecipitated ROR-γt using the cLPLs lysate from naive C57BL/6 mice for western blotting. The membrane was blotted with anti-HDAC2 antibody, revealing a physical interaction between ROR-γt and HDAC2 (Fig. 4a). We also tested whether the SUMOylation of ROR-γt facilitates its interaction with HDAC2. We immunoprecipitated ROR-γt using Th17 cells that expressed WT-ROR-γt or K187R-ROR-γt. The immunoprecipitated samples were blotted with anti-HDAC2 antibody, and we found that only WT-ROR-γt interacts with HDAC2 (Fig. 4b), confirming that SUMOylation of ROR-γt facilitates interaction with HDAC2. In contrast, analysis of SUMOylated ROR-γt interaction with HDAC1, which is known to interact with ROR-γt^23^, revealed that WT-ROR-γt and K187R-ROR-γt bind equally to HDAC1 (Supplementary Fig. 6b).

To confirm the physical interaction of HDAC2 with the IL-17 promoter, ChIP assays were performed on cLPLs from naive C57BL/6 mice using anti-HDAC2, anti-ROR-γt, or control antibody (IgG). As shown in Fig. 4c, HDAC2 engages the IL-17 promoter. To assess whether the SUMOylation of ROR-γt facilitates HDAC2 recruitment to the IL-17 promoter, ChIP assays were performed with Th17 cells that expressed WT-ROR-γt or K187R-ROR-γt. As shown in Fig. 4d, HDAC2 associates with the IL-17 promoter only in Th17 cells that express WT-ROR-γt but not the SUMOylation-deficient mutant of ROR-γt (K187R-ROR-γt). These findings support the view that SUMOylation of ROR-γt is necessary for binding of HDAC2 to the IL-17 promoter.

### HDAC2 inhibits IL-17 expression

**Fig. 5:**
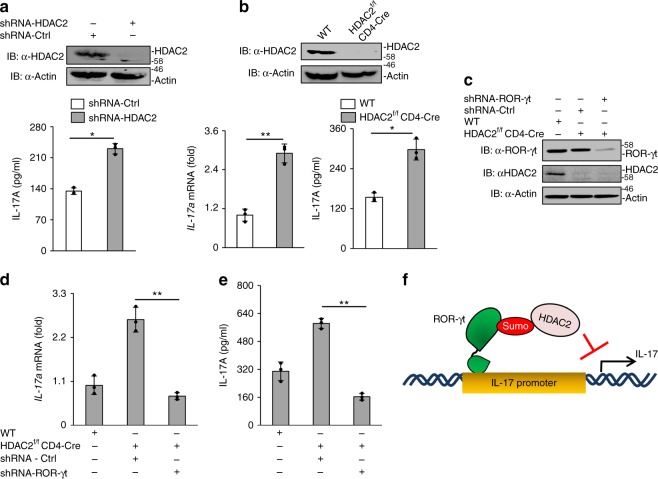
HDAC2 inhibits IL-17 expression. **a** Knockdown of HDAC2 in cLPLs of WT mice using shRNA. ELISA was performed to measure IL-17A secretion in the culture supernatant of WT cLPLs treated with HDAC2-specific shRNA (shHDAC2) or control shRNA (shCtrl). **b** CD4^+^ T cells were isolated from WT and HDAC2^f/f^ CD4-Cre mice and were differentiated under Th17-inducing conditions. Real-time PCR and ELISA were performed to measure IL-17A expression. **c** Immunoblot analysis was performed to check the knockdown of ROR-γt in CD4^+^ T cells isolated from HDAC2^f/f^ CD4-Cre mice using ROR-γt-specific shRNA and differentiated under Th17-inducing conditions. **d** ELISA and **e** real-time PCR were performed to measure IL-17A expression in HDAC2^f/f^ CD4-Cre cells treated with ROR-γt-specific shRNA or control shRNA. **f** Schematic drawing of model shows that SUMOylation of ROR-γt facilitates recruitment of HDAC2, which inhibits IL-17 expression. Data are from one experiment representative of three or more independent experiments with similar results. *p *<* 0.05 and **p *<* 0.01, ns non-significant (two-tail *t* test) error bars are S.D.

Next, IL-17-promoter-driven luciferase assays were performed to assess the model that ROR-γt SUMOylation inhibits IL-17 expression by facilitating the recruitment of HDAC2 to the IL-17 promoter. Using the Jurkat T cell model, WT-ROR-γt or K187R-ROR-γt expression vectors were transfected along with HDAC2 and the pGL4-mIL-17 promoter construct. On the following day, cultures were stimulated with PMA and ionomycin and harvested to measure luciferase activity. As shown in Fig. 4e, luciferase activity was inhibited when HDAC2 was expressed with WT-ROR-γt, but not when HDAC2 was expressed with the K187R-ROR-γt mutant, consistent with the possibility that SUMOylation of ROR-γt recruits HDAC2 to the IL-17 promoter and represses IL-17 expression. For confirmation, we used a catalytically inactive HDAC2 mutant in which a conserved histidine is replaced with alanine (HDAC2-H141A)^24^. Expression of the HDAC2-H141A mutant did not inhibit IL-17-promoter-driven luciferase activity (Fig. 4e), indicating that the catalytic activity of HDAC2 was essential for inhibition of ROR-γt transcriptional activity. Furthermore, shRNA-mediated knockdown of HDAC2 in cLPLs resulted in significant increases in IL-17A expression (Fig. 5a). Finally, we generated CD4^+^ T cell-specific conditional HDAC2 knockout mice (floxed-HDAC2 plus CD4-cre alleles). Flow cytometric analysis did not show any significant difference in the numbers of CD4^+^ or CD8^+^ T cells between the spleen, lymph node, and thymus of WT and HDAC2^f/f^ CD4-Cre mice. Similarly, the percentages of CD4^+^CD25^+^Foxp3^+^ T cells, naïve cells, and effector memory cells were comparable between HDAC2^f/f^ CD4-Cre and WT mice (Supplementary Fig. 7a–d). Moreover, HDAC2 deficiency did not affect T cell proliferation or survival (Supplementary Fig. 8a–b). We then isolated naive CD4^+^ T cells from HDAC2^f/f^ CD4-Cre mice and C57BL/6 mice that were differentiated under Th17-inducing conditions and checked for IL-17 expression by ELISA and real-time PCR (Fig. 5b). We observed markedly more IL-17A expression in the HDAC2^f/f^ CD4-Cre Th17 cells than in the WT Th17 cells. HDAC2 deficiency did not affect the differentiation of T cells into Th1 and Tregs, although a moderate increase in IL-4 production was detected in HDAC2^f/f^ CD4-Cre Th2 cells (Supplementary Fig. 9a–c).

To gain further evidence for HDAC2-mediated regulation of ROR-γt, we knocked down ROR-γt in HDAC2^f/f^ CD4-Cre CD4^+^ T cells using shRNA. The cells were then differentiated under Th17-inducing conditions, and the level of IL-17 expression was analyzed by real-time PCR and ELISA. As shown in Fig. 5c–e, ROR-γt depletion substantially attenuated IL-17A expression. Thus, SUMOylation of ROR-γt inhibits IL-17 expression via HDAC2 (Fig. 5f).

The central role of IL-17 has been highlighted in several human inflammatory diseases including inflammatory bowel diseases, rheumatoid arthritis, and multiple sclerosis^3,25,26^. To gain in vivo evidence for HDAC2-mediated regulation of IL-17-mediated inflammation, we adoptively transferred WT or HDAC2^f/f^ CD4-Cre CD4^+^CD45RB^hi^ cells into Rag1^–/–^ mice. HDAC2^f/f^ CD4-Cre cells caused greater loss of body weight, higher diarrhea score, and a higher weight-to-length ratio of the colon than WT cells (Supplementary Fig. 10a–d). As expected, the expression of IL-17A was higher in the colonic mucosa of Rag1^–/–^ mice that received HDAC2^f/f^ CD4-Cre cells than in mice that received WT cells (Supplementary Fig. 10e). Histological analysis showed greater infiltration of inflammatory cells, more crypt damage, and higher clinical scores for Rag1^–/–^ mice that received HDAC2^f/f^ CD4-Cre cells than those that received WT cells (Supplementary Fig. 10f–g). Thus, HDAC2^f/f^ CD4-Cre CD4^+^CD45RB^hi^ cells are highly colitogenic.

**Fig. 6:**
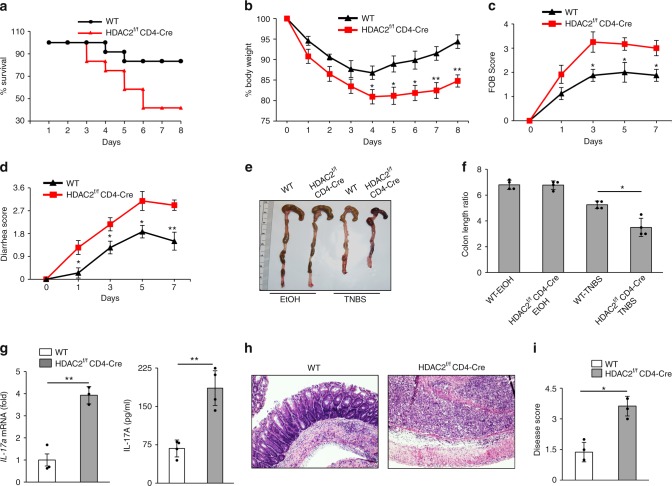
HDAC2^f/f^ CD4-Cre mice show enhanced colitis. Data shows **a** survival curve (%) (*n* *=* 12 per group), **b** percentage change in body weight, **c** FOB score, **d** diarrhea score, and **e**, **f** colon length of WT mice and HDAC2^f/f^ CD4-Cre mice (*n* *=* 6 per group) after intrarectal administration of 2.5% TNBS. **g** Real-time PCR and ELISA were used to analyze IL-17A expression in WT mice and HDAC2^f/f^ CD4-Cre mice given TNBS. **h** H&E-stained colonic sections and **i** disease scores of those sections are shown from WT mice and HDAC2^f/f^ CD4-Cre mice as in **a**–**d** (*n* = 4). Data are from one experiment representative of three independent experiments with similar results. *p *<* 0.05 and **p *<* 0.01 (two-tail *t* test) error bars are S.D.

Further, we induced colitis in HDAC2^f/f^ CD4-Cre mice using the 2, 4, 6-trinitrobenzenesulfonic acid (TNBS) model, where IL-17 plays a critical role^27–29^. We administrated TNBS intrarectally into HDAC2^f/f^ CD4-Cre mice, and the severity of colitis was compared with that of WT mice. As shown in Fig. 6a, we found increased mortality in HDAC2^f/f^ CD4-Cre mice compared to WT control. We found severe body weight loss, rectal bleeding, and diarrhea among HDAC2^f/f^ CD4-Cre mice (Fig. 6b–d). Also, HDAC2^f/f^ CD4-Cre mice had shorter and thicker colons than WT mice (Fig. 6e, f). There was also increased expression of *IL-17a* mRNA in the colonic mucosa and increased IL-17A secretion in the explant colon culture of HDAC2^f/f^ CD4-Cre compared to WT mice (Fig. 6g). No significant difference in the level of *IL-2*, *IFN-γ*, and *IL-4* was observed (Supplementary Fig. 11). Histological examination showed increased infiltration of inflammatory cells, more crypt damage, and higher disease scores in the HDAC2^f/f^ CD4-Cre mice (Fig. 6h–i).

To expand our understanding of the role of HDAC2-mediated regulation of IL-17-driven inflammation in vivo, we extended our studies to include experimental autoimmune encephalomyelitis (EAE), a mouse model of human multiple sclerosis. We immunized WT and HDAC2^f/f^ CD4-Cre mice with the myelin oligodendrocyte glycoprotein (MOG_35-55_) peptide in complete Freund’s adjuvant. HDAC2^f/f^ CD4-Cre mice developed EAE faster and with greater severity than WT mice, as indicated by disease scores (Supplementary Fig. 12a). As expected, the IL-17A level was significantly higher in spinal cord as well as cultured splenocytes of HDAC2^f/f^ CD4-Cre mice (Supplementary Fig. 12b–c). No significant difference in the level of *IL-2*, *IFN-γ*, and *IL-4* was observed (Supplementary Fig. 12d–f). Histological analysis of H&E-stained sections showed increased infiltration of inflammatory cells into the spinal cords of HDAC2^f/f^ CD4-Cre mice compared to WT mice (Supplementary Fig. 12g–h). Together, these data strongly support a role for HDAC2 in the repression of IL-17 expression and pathogenic autoimmune inflammation.

## Discussion

SUMOylation is an evolutionarily conserved post-translational modification that has a central regulatory role in eukaryotic cells^9–11^, but its function is not well studied in immune cells. Here, we show that SUMOylation represses IL-17 gene expression, which encodes a major proinflammatory cytokine. The SUMO-conjugating enzyme Ubc9 recognizes a conserved GKAE motif within IL-17-inducing transcription factor ROR-γt. CD4^+^ T cells expressing SUMOylation-defective ROR-γt produce elevated levels of IL-17 and are highly colitogenic upon transfer to Rag1^−/−^ mice. SUMOylated ROR-γt recruits HDAC2 to the IL-17 promoter to repress gene expression. Our findings show a Ubc9–ROR-γt-IL-17 pathway that serves a critical role in the regulation of IL-17 expression and hence IL-17-mediated inflammation.

While robust IL-17 responses are essential for the clearance of pathogens, chronic IL-17 responses can provoke various diseases including psoriasis, rheumatoid arthritis, spondylitis, multiple sclerosis, and inflammatory bowel diseases^2,30^. Our studies show that ROR-γt is the master transcription factor that controls IL-17 expression, making this molecule an attractive drug target for precisely controlling IL-17-mediated immunity^8^. Prior studies have focused on how ROR-γt expression is regulated and its role in differentiation of Th17 and innate lymphoid cells. However, there is a limited understanding of how ROR-γt function to modulate IL-17 expression is spatiotemporally regulated in response to inflammatory stimuli without causing excessive inflammation.

Our study uncovers a detailed mechanism that attenuates IL-17 expression via ROR-γt SUMOylation, which holds therapeutic potential for clinically boosting IL-17 immunity against pathogens or preventing pathological Th17-mediated autoimmunity by controlling the ROR-γt/IL-17 pathways.

Recent reports have provided insights into the regulation of ROR-γt protein stability by the ubiquitin-proteasome pathway^7,31^. We showed earlier that the E3 ubiquitin ligase Itch promotes ROR-γt degradation by ubiquitinating it^12^. The deubiquitinases USP17 and USP4 have been shown to stabilize ROR-γt by decreasing ROR-γt ubiquitination^32,33^. Additionally, DUBA (OTUD5), another deubiquitinase, has also been shown to regulate ROR-γt protein stability. Although cross-talk between ubiquitination and SUMOylation is known^9^, our studies showed no apparent effect of SUMOylation on ROR-γt protein stability, suggesting distinct regulatory roles for ubiquitination and SUMOylation of ROR-γt. However, we do not exclude the possibility of other modification, including acetylation or methylation, at K187 of ROR-γt.

In addition to Ubc9, SUMOylation in vivo requires SUMO-E3 ligases, which increase the rate of SUMO conjugation^9–11^. In addition to PIAS1, PIAS3, PIASxα, PIASxβ, PIASy, and RanBP2^9–11^, certain TRIM family members can act as SUMO-E3 ligases that utilize Ubc9 as an E2^34^. At present, which SUMO E3 ligase promotes ROR-γt SUMOylation remains unclear. Also, SUMOylation is a reversible process and is regulated by SUMO-specific proteases (SENPs) that include SENP1, SENP2, SENP3, SENP5, SENP6, SENP7, and SENP8^35^. Whether these SENPs are involved in deSUMOylation of ROR-γt requires further investigation.

Histone acetylation by acetyl transferases and deacetylation by HDACs play a critical role in regulating chromatin structure and gene expression^36^. It has been shown that HDAC1 binds to ROR-γt and reciprocally regulates IL-17 expression with the acetylase p300^23^. Our studies show that the binding of HDAC1 is independent of ROR-γt SUMOylation, which suggests a distinct role for HDAC1 and HDAC2 in regulating ROR-γt transcriptional activity. Our results also show that CD4^+^ T cell-specific conditional deletion of HDAC2 exacerbates colitis and EAE, and this is associated with elevated IL-17 expression. These results are in line with a recent report that HDAC2^-/+^ (heterozygous) mice developed severe airway inflammation that was induced by cigarette smoke, and deletion of IL-17A attenuated smoke-induced airway remodeling in these mice^37^. However, we do not exclude the possibility of defect in acetylation of ROR-γt or other factors that may contribute to the observed phenotype in HDAC2^f/f^ CD4-Cre mice.

In summary, our study has revealed a detailed mechanism by which IL-17-mediated inflammation is regulated by SUMOylation of ROR-γt. Further exploration of this pathway can expand our knowledge of post-translational regulation of immune cell function and help to devise strategies to treat inflammatory diseases.

## Methods

### Mice

C57BL/6, Rag1^−/−^, and Rorctm1Litt/J ROR^−/−^ mice were purchased from Jackson Laboratory, as were HDAC2^f/f^ mice^38^. CD4^+^ T cell-specific conditional HDAC2 knockout mice were generated by crossing CD4-Cre mice with HDAC2^f/f^ mice. All mice were housed in microisolator cages in the barrier facility of Baylor Institute for Immunology Research. All experiments were performed in accordance with the guidelines of the Institutional Animal Care and Use Committee of Baylor Research Institute.

### Plasmids, antibodies, and reagents

The plasmid Flag-mROR-γt was created from MIGR-mROR-γt (#24069; Addgene) and cloned into pCMV-Tag2B vector. Flag-K187R-mROR-γt was generated by site-directed mutagenesis using Flag-mROR-γt as a template. Myc-Ubc9 (#20082), HA-SUMO1 (#21154), SUMO2 (#48967), SUMO3 (#17361), HDAC2 (#68117), and pGL4-mIL-17pr (#20124) were purchased from Addgene (Cambridge, MA, USA). Myc-Foxp3 and Myc-Runx1 plasmids were generated by using the templates MIGR-mFoxp3 (#24067, Addgene) and HA-Runx1 (#45815, Addgene), respectively. Myc-Ubc9-C93A and Flag-HDAC2-H141A plasmids were generated by mega primer approach. Lentiviral expression clones of mROR-γt and K187R-mROR-γt were constructed by first subcloning into the pENTR-3C entry vector (Invitrogen, Carlsbad, CA, USA) and then L-R recombined to pLenti6.2/N-Lumio/V5-DEST. The sequences of all clones were verified. Antibodies used in these studies were anti-c-Myc (1:1000; #9E10, Santa Cruz, Dallas, TX, USA), anti-HDAC1 (1:1000; #10E2, Cell Signaling Technology, Danvers, MA, USA), anti-HDAC2 (1:1000; #C-8, Santa Cruz), anti-SUMO1 (1:500; #FL-101, Santa Cruz), anti-HDAC2 (#ab12169, Abcam, Cambridge, UK) for ChIP assay, anti-Flag (1:3000; #M2, Sigma, St. Louis, MO, USA), anti-β-actin (1:2000; #AC-15, Sigma), anti–ROR-γt (1:800; BD Bioscience, Franklin Lakes, NJ, USA), anti–ROR-γt (clone #AKFS9, eBioscience, San Diego, CA, USA) for ChIP assay, anti-hemagglutinin (HA) (1:1000; #Y-11, Santa Cruz), V5 antibody (1:4000; #R960-25, Invitrogen), and Clean-Blot^TM^ IP detection reagent (HRP) (1:50; Pierce Biotechnology, Waltham, MA, USA). Resazurin Sodium Salt (#R7017) was from Sigma-Aldrich (St. Louis, MO, USA) and the CFSE cell division tracker kit (#423801) was from Biolegend (San Diego, CA, USA). Fc block (#553142, BD Bioscience), Live Dead Aqua (#L34957, Invitrogen), anti-CD4-FITC (clone GK1.5, #11-0041-85, eBioscience), anti-CD8-PerCp-cy5.5 (clone 53-6.7, #551162, BD Pharmingen, Franklin Lakes, NJ, USA), anti-CD44-FITC (clone IM7, #553133, BD Pharmingen), anti-CD62L-APC-efluor^TM^ 780 (clone MEL-14, #47-0621-82, eBioscience), anti-CD4-PE (clone GK1.5, #553730, BD Pharmingen), anti-CD25-PE (clone PC61.5, #12-0251-81, eBioscience), CD4-APC (Clone GK1.5, #17-0041-81, eBioscience), and Foxp3-PE (FJK-16s, #12-5773-80, eBioscience) were used for flow cytometry experiments. Ready-SET-Go! ELISA kit for mouse IL-17 was purchased from eBioscience. The Dual-Luciferase Reporter Assay System kit was purchased from Promega (Madison, WI, USA). The Amaxa Cell Line Nucleofector kit was purchased from Lonza (Basel, Switzerland). Ubc9 (sc-36774-V), ROR-γt (sc-38881-V), and HDAC2 (sc-29346-V) shRNA lentiviral particles were purchased from Santa Cruz Biotechnology. Anti-CD28 (clone-37.51, #102101), anti-CD3 (clone-145-2C11, #100313), anti-IL4 (clone-11B11, #504102), and anti-IFN-γ (clone-XMG1.2, #505802) were purchased from BioLegend. Recombinant murine IL-6 (#216-16-B), recombinant murine IL-12 (#212-12), recombinant human TGF-β (#100-21), and recombinant murine IL-4 (#214-14) were purchased from PeproTech.

### Cell culture and transfection assay

293 T and 293FT cells (R700-07) were purchased from Invitrogen, and Jurkat T cells were purchased from ATCC. The medium used for these cell lines was Dulbecco’s modified Eagle’s medium supplemented with 10% fetal bovine serum. Transfections were carried out using Lipofectamine 2000 (Invitrogen), except for Jurkat T cells, which underwent electroporation with the Amaxa kit according to the manufacturer’s instructions. The luciferase assay was performed using the luciferase reporter gene assay kit (Promega) according to the manufacturer’s instructions. One day before transfection, 5 × 10^5^ cells were seeded in 6-well culture plates, and the assay was performed after 30 h of transfection.

### CD4^+^ T cell differentiation and lentiviral transduction

CD4^+^ T cells were purified from splenocytes by magnetic activated cell sorting beads according to the manufacturer’s protocol (Miltenyi Biotec, Bergisch Gladbach, Germany). The purified CD4^+^ T cells were transduced with lentivirus expressing WT-ROR-γt or K187R-ROR-γt and differentiated under Th17 polarizing conditions The cells were then restimulated with 50 ng ml^-1^ PMA and 1 μg ml^−1^ ionomycin for 4 h, and ELISA was performed from culture supernatants. For differentiation, 3 ng ml^−1^ of IL-12 was used for Th1, 10 ng ml^−1^ of IL-4 for Th2, 1 μg ml^−^^1^ of α-IFN-γ, and 5 ng ml^−1^ of TGF-β for Treg, and culture for 5–7 days.

### Luciferase assay

Jurkat T cells were transfected with Ubc9, SUMO1, the IL-17 promoter plasmid, and either WT-ROR-γt or K187R-ROR-γt using the Amaxa Cell Line Nucleofector kit. After 24 h of transfection, cells were stimulated with PMA (50 ng ml^−1^) and ionomycin (1 μg ml^−1^). Lysates were prepared using the Dual-Luciferase Reporter Assay System kit, and luminescence was measured.

### Immunoblot analysis and immunoprecipitation

Cells were lysed in a NP-40 lysis buffer (50 mM Tris–HCl, pH 7.4, 150 mM NaCl, 1% NP-40, supplemented with cocktail protease inhibitor). Protein estimations were done using the Pierce BCA protein assay kit according to the manufacturer’s protocol. Protein samples were resolved on SDS-PAGE and were transferred to the polyvinylidene difluoride (PVDF) membrane by a wet blot system (Bio-Rad, Hercules, CA, USA). Post-transfer, the membrane was transferred to a blocking buffer (phosphate-buffered saline, 5% skimmed milk, and 0.1% Tween-20) for 1 h at room temperature. After incubation, the blot was washed 3 times (10 min each) with washing buffer (Tris-buffered saline and 0.1% Tween-20). Subsequently, the membrane was incubated overnight at 4 °C with primary antibodies diluted in blocking buffer followed by washing 3 times (10 min each) with washing buffer. The membrane was then incubated with secondary antibodies conjugated to poly-horseradish peroxidase (HRP) for another 1 h at room temperature. After subsequent washing, a blot was developed with ECL (Amersam, Little Chalfont, UK) western blotting detection reagents. For immunoprecipitation, cells were harvested and lysed in NP-40 buffer at 4 °C for 20 min. After centrifugation, supernatant was transferred to fresh tubes. Approximately 10% of the whole-cell lysate was used as input. Whole-cell lysates were precleared with 20 μL of Protein A/G plus agarose beads (Millipore, Billerica, MA, USA) for 1 h at 4 °C. Lysates were then incubated with 1 μg of the desired antibody overnight at 4 °C followed by 1 h incubation at 4 °C with 25 μL of Protein A/G beads. The immunocomplexes were washed 5 times with NP-40 buffer, denatured using 4× Laemmli buffer, separated by SDS-PAGE, transferred to PVDF membranes, and analyzed by immunoblotting. Clean-Blot^TM^ IP detection reagent (HRP) was used as a secondary antibody in all immunoprecipitation assays.

### Protein identification by liquid chromatography-tandem MS

Total lysates were prepared from cLPLs of WT mice followed by immunoprecipitation with anti-ROR-γt antibody or control antibody (IgG). Immunoprecipitated proteins were separated by SDS-PAGE. In-gel digestion with trypsin, followed by protein identification using liquid chromatography-tandem MS, was performed. Briefly, tryptic peptides were resolved on a nano-liquid chromatography column (Magic AQ C18; Michrom Bioresources, Auburn, CA, USA) and introduced into an Orbitrap mass spectrometer (Thermo Scientific, Waltham, MA, USA). The Orbitrap was set to collect a high-resolution MS1 (FWHM 30,000@400 m/z), followed by the data-dependent collision-induced dissociation spectra on the “top 9” ions in the linear ion trap. Spectra were searched against a human protein database (UniProt release 2011_05) using the X!Tandem/TPP software suite^39^. Proteins identified with a Protein Prophet probability ≥ 0.9 and a false discovery rate <2% were considered for further analysis.

### Ubiquitination assay

293 T cells were transfected with either Flag-WT-ROR-γt or Flag-K187R-ROR-γt along with Myc-Itch and HA-Ub. MG132 was added 4 h before cell lysis. Cells were washed 3 times with phosphate-buffered saline and lysed in NP-40 lysis buffer. Immunoprecipitation was performed using anti-Flag antibody. ROR-γt-associated ubiquitin was analyzed by immunoblot using anti-HA antibody.

### SUMOylation assay

To assess SUMOylation of ROR-γt, 293 T cells expressing HA-SUMO1, Myc-Ubc9, and Flag-ROR-γt or Flag-K187R-ROR-γt were lysed in lysis buffer (NP-40 lysis buffer containing 20 mM N-ethylmeimide, 1% SDS) and incubated for 10 min at 95 °C. Finally, samples were diluted with NP-40 lysis buffer containing 20 mM *N*-ethylmaleimide prior to immunoprecipitation. Protein immunoprecipitation studies were performed using anti-Flag antibody and immunoblotted with anti-HA antibody to detect the SUMOylated form of ROR-γt.

### RNA-Seq library preparation and data analysis

RNA was isolated from Th17 cells expressing either WT-ROR-γt or a SUMO-deficient mutant of ROR-γt using Qiagen RNA easy mini kit. Poly-A enriched NGS library construction was performed using the KAPA mRNA HyperPrep Kit (KAPA Biosystems, Wilmington, MA, USA) according to the manufacturer’s protocol. Quality of the individual libraries was assessed using the High Sensitivity DNA Kit (Agilent, Santa Clara, CA, USA). Individual libraries were quantitated via qPCR using the KAPA Library Quantification Kit, Universal (KAPA Biosystems) and equimolar pooled. Final pooled libraries were sequenced on an Illumina NextSeq 500 with paired-end 75 bases read lengths. Sequencing read quality was evaluated using FastQC. Reads after quality and adapter trimming by Cutadapt were aligned with HiSat2 to GRCm38. Read counts were generated by the featureCounts program using the annotations from GENCODE M15. Differential gene expression analysis was performed using DESeq2.

### Real-time PCR analysis

Total RNA was prepared using the RNeasy Mini kit (Qiagen, Hilden, Germany) followed by cDNA synthesis using the Verso cDNA Kit (Thermo Scientific, Waltham, MA, USA). Quantitative real-time PCR was performed on a Mastercycler Realplex2 (Eppendorf, Hamburg, Germany) using lightCycler 480 SYBR-Green Master Mix (Roche, Basel, Switzerland). All reactions were completed in triplicate. The expression of individual genes was normalized to the expression of actin. The following cycling parameters were used: 95 °C for 2 min, followed by 40 cycles of 95 °C for 15 s, an annealing temperature of 55 °C for 15 s, and 72 °C for 20 s. The primer sequences for the genes are as follows:

IL-17A forward primer: 5ʹ-TTTAACTCCCTTGGCGCAAAA-3ʹ

IL-17A reverse primer: 5ʹ-CTTTCCCTCCGCATTGACAC-3ʹ

IL-17F forward primer: 5ʹ-CTGGAGGATAACACTGTGAGAGT-3ʹ

IL-17F reverse primer: 5ʹ-TGCTGAATGGCGACGGAGTTC-3ʹ

ROR-γt forward primer: 5ʹ-TACCTTGGCCAAAACAGAGG-3ʹ

ROR-γt reverse primer: 5ʹ-ATGCCTGGTTTCCTCAAAA-3ʹ

mIL23R forward primer: 5′-GCTCGGATTTGGTATAAAGG-3′

mIL23R reverse primer: 5ʹ- ACTTGGTATCTATGTAGGTAGG-3ʹ

mFoxp3 forward primer: 5ʹ-CCCATCCCCAGGAGTCTTG-3ʹ

mFoxp3 reverse primer: 5ʹ-ACCATGACTAGGGGCACTGTA-3ʹ

mIL-4 forward primer: 5ʹ-GGTCTCAACCCCCAGCTAG-3ʹ

mIL-4 reverse primer: 5ʹ-GCCGATGATCTCTCTCAAGT-3ʹ

mIL-2 forward primer: 5ʹ-GTGCTCCTTGTCAACAGCG-3ʹ

mIL-2 reverse primer: 5ʹ-GGGGAGTTTCAGGTTCCTGTA-3ʹ

mIFN-γ forward primer: 5ʹ-GAACTGGCAAAAGGATGGTGA-3ʹ

mIFN-γ reverse primer: 5ʹ-TGTGGGTTGTTGACCTCAAAC-3ʹ

β-Actin forward primer: 5ʹ-GAAATCGTGCGTGACATCAAAG-3ʹ

β-Actin reverse primer: 5ʹ-TGTAGTTTCATGGATGCCACAG-3ʹ

### Adoptive transfer of Th17 cells

The CD4^+^CD25–CD45RB^hi^ cells were transduced with lentivirus expressing either WT-ROR-γt or K187R-ROR-γt and cultured under Th17 conditions as described above. The lentiviral-transduced Th17 cells (5 × 10^5^ cells/mice) were injected intraperitoneally into 8-week-old Rag1^−/−^ mice, and the mice were monitored for body weight and diarrhea score up to 6 weeks.

### Chromatin immunoprecipitation assay

The cells were cross-linked with 1% (v/v) methanol-free formaldehyde for 10 min (histone modification) and processed according to the protocol described in the Chromatin Immunoprecipitation Assay Kit (Millipore). Antibody chromatin complexes were pulled down using protein A/G beads, washed, and then eluted. After cross-link reversal and proteinase K treatment, immunoprecipitated DNA was purified using the ChIP DNA Clean kit (Zymo Research, Irvine, CA, USA). PCR was carried out with appropriate primers. An equal amount of chromatin solution was precipitated. IgG antibody was used as a negative control. The primer sequences for PCR are as follows:

mIL-17 promoter forward primer: 5ʹ-GACAGATGTTGCCCGTCATA-3ʹ

mIL-17 promoter reverse primer: 5ʹ-CAACAAGCGCCTTGTACATTAG-3ʹ

### TNBS-induced colitis

Colitis was induced in 6-to-8-week-old mice by presensitizing their skin with 150 μl of 1% TNBS (Sigma, St Louis, MO) in an acetone/olive oil mix (4:1) followed by rectal administration of 2.5% TNBS in ethanol after 7 days of presensitization. Control mice were treated with 50 μl of 50% ethanol alone. Body weight was measured every day and calculated as percent change in weight compared to baseline. Mice were killed on day 8 after rectal administration of TNBS. Stool consistency, occult blood, and histology were scored. Briefly, stool scores were calculated as follows: 0 = well-formed pellets, 2 = semiformed stool, and 4 = liquid stool that adhered to the anus. Bleeding scores were calculated as follows: 0 = no blood, 2 = visible blood traces in stool, and 4 = gross rectal bleeding. Stool consistency scores and bleeding scores were added and presented as clinical scores. Histology were calculated as follows: 0 = no evidence of inflammation, 1 = low level of inflammation, with scattered infiltrating mononuclear cells, 2 = moderate inflammation, 3 = high level of inflammation, with increased vascular density and marked wall thickening, 4 = maximal severity of inflammation with complete crypt loss.

### Cell proliferation assay

Purified CD4^+^ T cells were labeled with 5 μM CFSE for 10 min at 37 °C and cultured in the presence of α-CD3 and α-CD28 antibodies for 72 h. Cell proliferation was analyzed by FACS according to CFSE dilution and cell number counts. Cell viability was measured using Resazurin Sodium Salt (#R7017, Sigma). Purified CD4^+^ T cells were incubated 1-4 h with 0.01% Resazurin Sodium Salt and fluorescence intensity was measured.

### Experimental encephalitis model

Wild type (WT) and HDAC2^f/f^ CD4-Cre mice 10–12 weeks of age were immunized subcutaneously on day 0 with 100 μg MOG (35–55) peptide. Pertussis toxin in 100 μl saline was injected subcutaneously twice (once each on days 0 and 1). Disease severity was assigned scores on the following scale: 0, no disease; 0.5, stiff tail; 1, limp tail; 1.5, limp tail with inability to right; 2, paralysis of one limb; 2.5, paralysis of one limb and weakness of another limb; 3, complete paralysis of both hind limbs; 4 moribund; 5, death.

### T cell–induced colitis

Splenocytes from WT and HDAC2^f/f^ CD4-Cre mice were enriched for CD4^+^ T cells by using the CD4^+^ T Cell Isolation Kit (MACS Miltenyi Biotech). Cells were then stained with antibodies against PE–CD45RB (16 A) and FITC-CD4 (GK1.5) (all from BD Pharmingen) and were then sorted for CD4^+^CD45RB^hi^ populations. Rag1^–/–^ mice were injected intraperitoneally with 5 × 10^5^ CD4^+^CD45RB^hi^ cells and monitored for body weight and diarrhea score up to 6 weeks.

### Densitometry analysis

To measure the levels of SUMOylated protein, the intensity of SUMOylated and unSUMOylated bands was quantified by densitometry using ImageJ version 1.43 software (National Institutes of Health, Bethesda, MD, USA). We considered the densitometry values of SUMOylated and unSUMOylated areas as 100% protein. From this value, we then calculated the percentage of SUMOylated and unSUMOylated proteins.

### Statistical analysis

The data were analyzed with GraphPad Prism 4 software (La Jolla, CA, USA) to determine statistical significance using the paired Student’s *t*-test. The data are expressed as mean ± S.D. A *p*-value < 0.05 was considered significant.

## Electronic supplementary material

Supplementary Information

## Data Availability

The raw data for RNAseq analysis have been deposited in the GEO data base under accession code GSE116407. The authors declare that all data supporting the findings of this study are available within the article and its supplementary file or from the corresponding author upon reasonable request.
