# Supplementary material for: SUMOylation of ROR-γt inhibits IL-17 expression and inflammation via HDAC2
Source: Nat Commun. 2018 Oct 30;9:4515. doi: 10.1038/s41467-018-06924-5 (PMC6207785; doi:10.1038/s41467-018-06924-5)

Supplementary Information

SUMOylation of ROR-γt inhibits IL-17 expression and inflammation via HDAC2

Singh et al.

Supplementary Figures

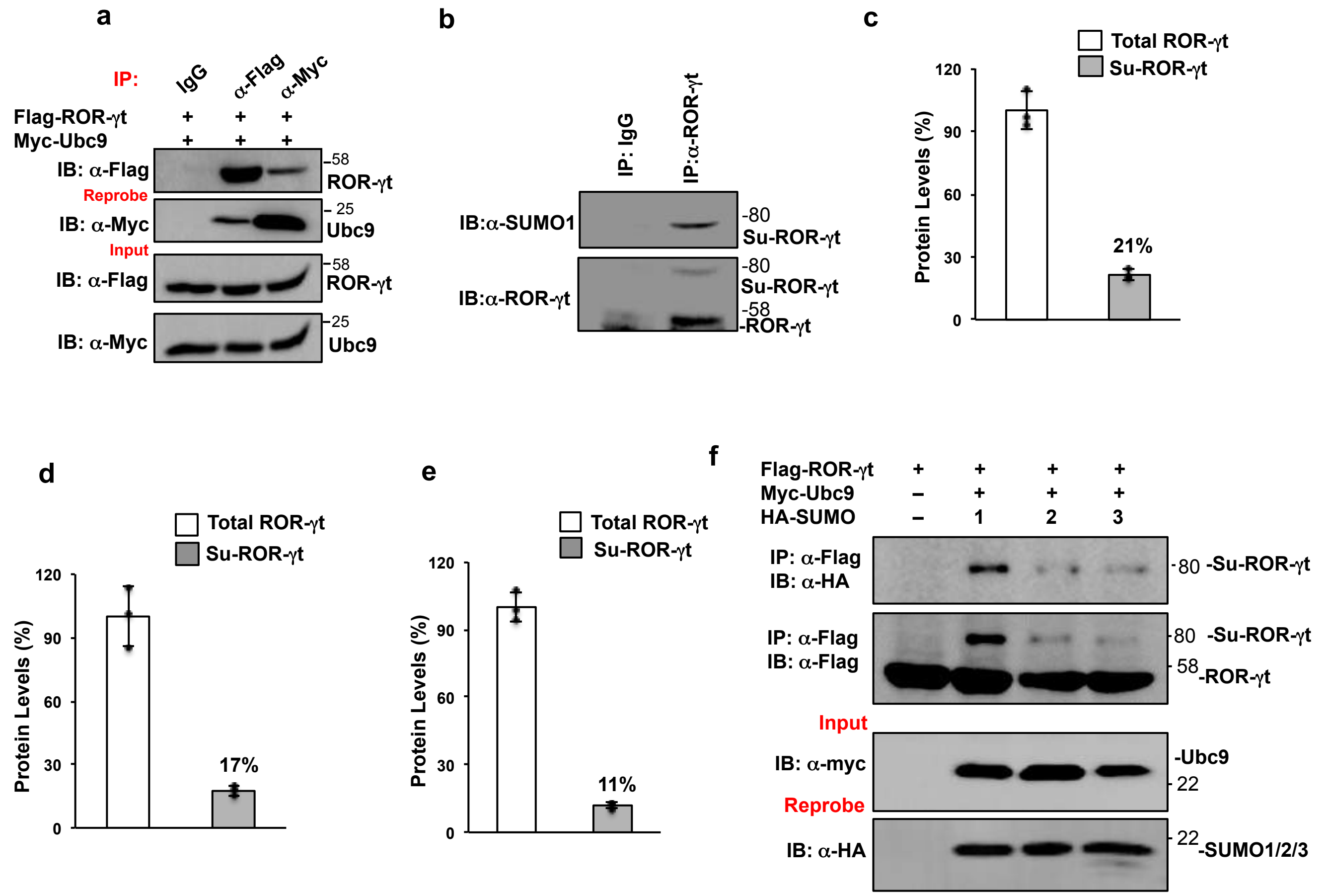

Supplementary Figure 1. Analysis of SUMOylation of ROR-γt. **(a)** Whole-cell lysates of 293T cells transfected with plasmids encoding Flag-ROR-γt and Myc-Ubc9 were immunoprecipitated (IP) with anti-c-Myc, anti-Flag, or the control antibody (IgG), and immunoblot analysis (IB) with anti-c-Myc or anti-Flag was performed. **(b)** Lysates were prepared from Th17 cells and immunoprecipitated with anti-ROR-γt antibody or control IgG antibody. The immunoprecipitates were analyzed by immunoblotting with anti-SUMO1 antibody. (**c**–**e)** Densitometry analysis was conducted for SUMOylated ROR-γt in **(c)** 293T cells, (**d)** cLPLs, and **(e)** in vitro differentiated Th17 cells. **(f)** 293T cells were transfected with Flag-ROR-γt and Myc-Ubc9 along with HA-SUMO1, HA-SUMO2, or HA-SUMO3. Cell lysates were immunoprecipitated with anti-Flag antibody. The immunoprecipitates were analyzed by immunoblot assay with anti-HA antibody to detect the SUMOylated form of ROR-γt. Data are representative of three independent experiments.

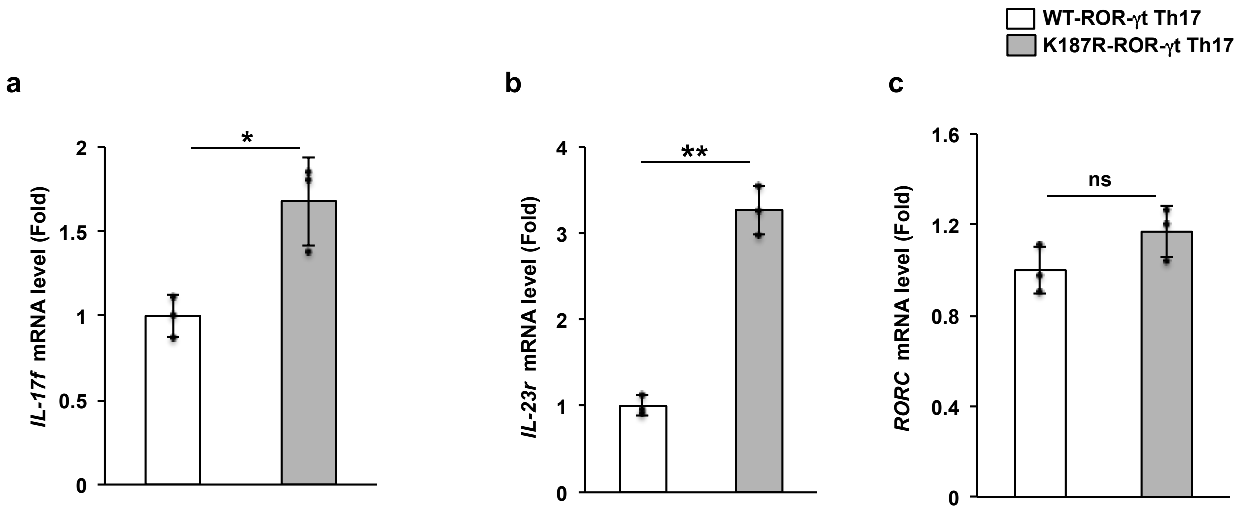

Supplementary Figure 2. Elevated levels of IL-17 expression in Th17 cells expressing a SUMOylation-deficient mutant of ROR-γt. **(a-c)** Real-time PCR analysis for *IL-17f*, *IL-23r,* and *RORC* genes. Data are from one experiment representative of four independent experiments with similar results. *$p < 0.05$, **$p < 0.01$ (two-tail *t* test) error bars are S.D.

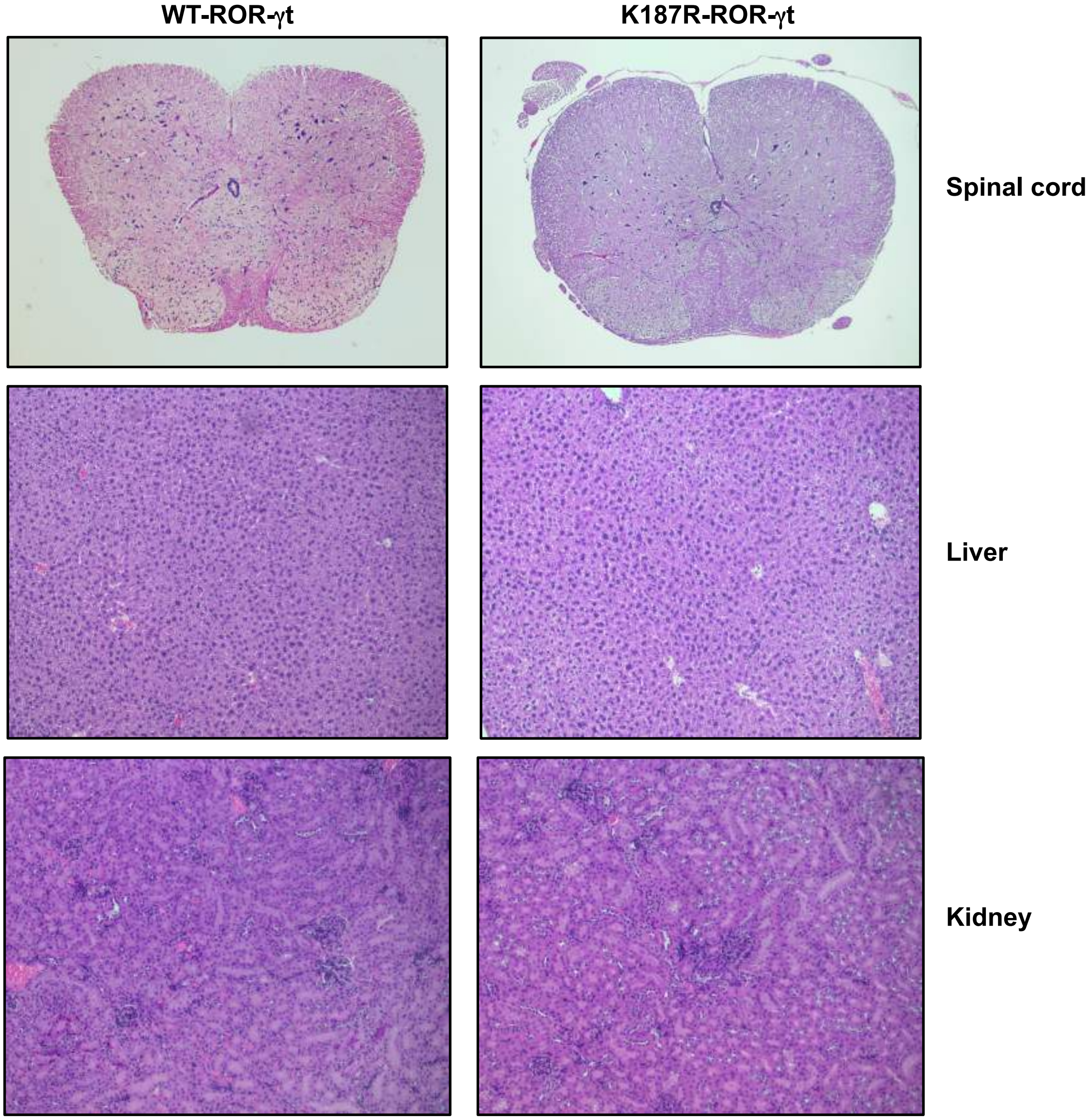

Supplementary Figure 3. Liver, kidney, and spinal cord of Rag1$^{-/-}$ mice injected with Th17 cells. H&E-stained sections of spinal cord, liver, and kidney were analyzed under a light microscope. Data are from one experiment representative of three independent experiments with similar results.

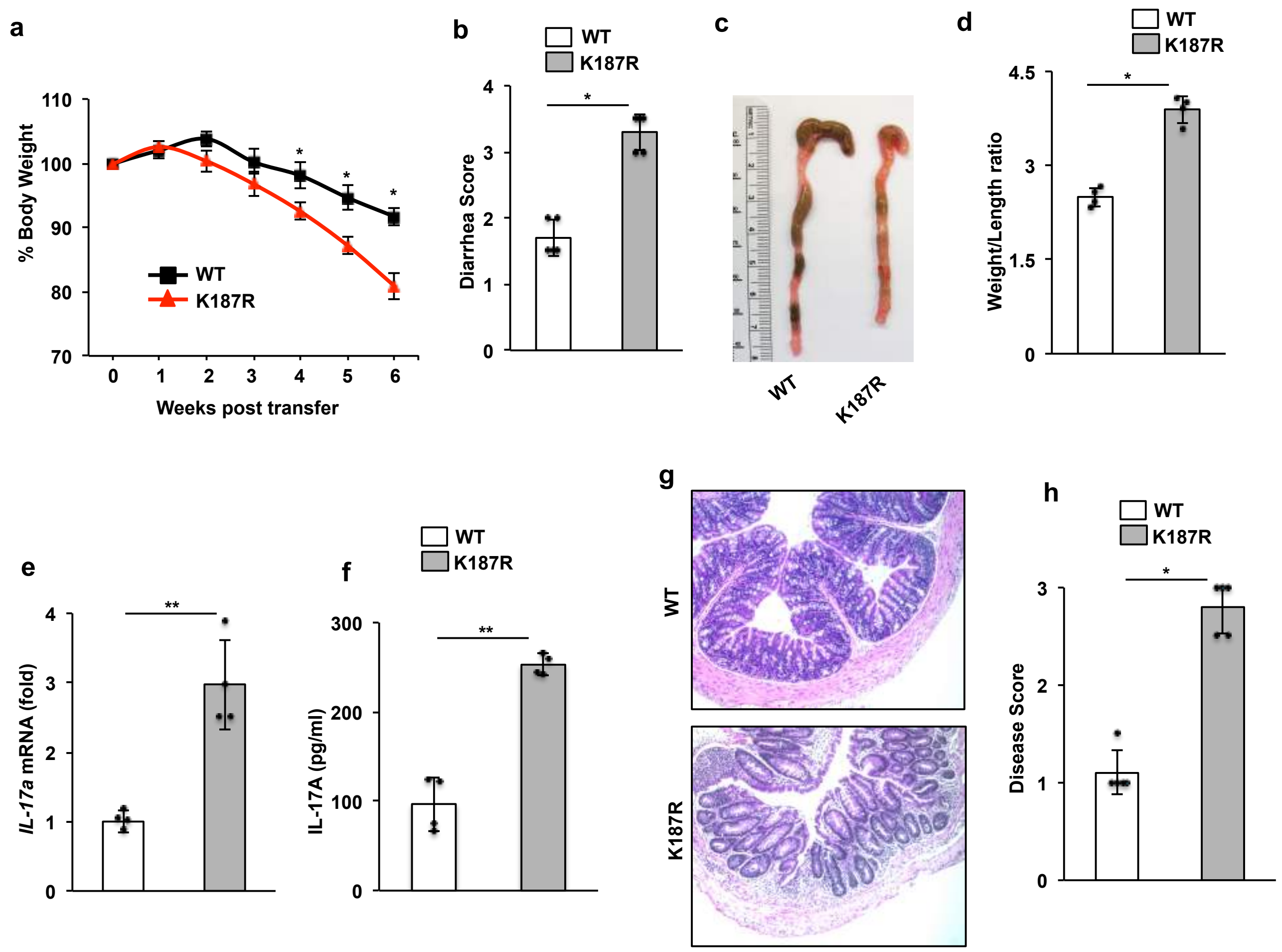

Supplementary Figure 4. Undifferentiated CD4$^+$ T cells expressing SUMOylation-defective ROR-γt induce severe colitis in Rag1$^{-/-}$ mice. ROR-γt$^{-/-}$ CD4$^+$ T cells transduced with lentivirus expressing WT-ROR-γt or SUMOylation-deficient mutant ROR-γt were adoptively transferred to Rag1$^{-/-}$ mice. Data show **(a)** body weight, **(b)** diarrhea score, **(c)** colon size, and **(d)** weight-to-length ratio of colon for Rag1$^{-/-}$ host mice (n = 4 per group) given an intraperitoneal injection of ROR-γt$^{-/-}$-transduced cells expressing WT-ROR-γt or SUMOylation-defective ROR-γt (K187R-ROR-γt) and monitored for 6 weeks. **(e)** Real-time PCR analysis was conducted of *IL-17a* mRNA as well as **(f)** ELISA to measure IL-17A secretion in the explant colon culture of Rag1$^{-/-}$ mice as in **a-d**. Results are presented relative to those of Rag1$^{-/-}$ mice given cells expressing WT-ROR-γt. **(g)** H&E-stained colonic sections and **(h)** disease score of those sections from Rag1$^{-/-}$ mice as in **a-d.** Data are from one experiment representative of three independent experiments with similar results. *$p < 0.05$, **$p < 0.01$ (two-tail t test) error bars are S.D.

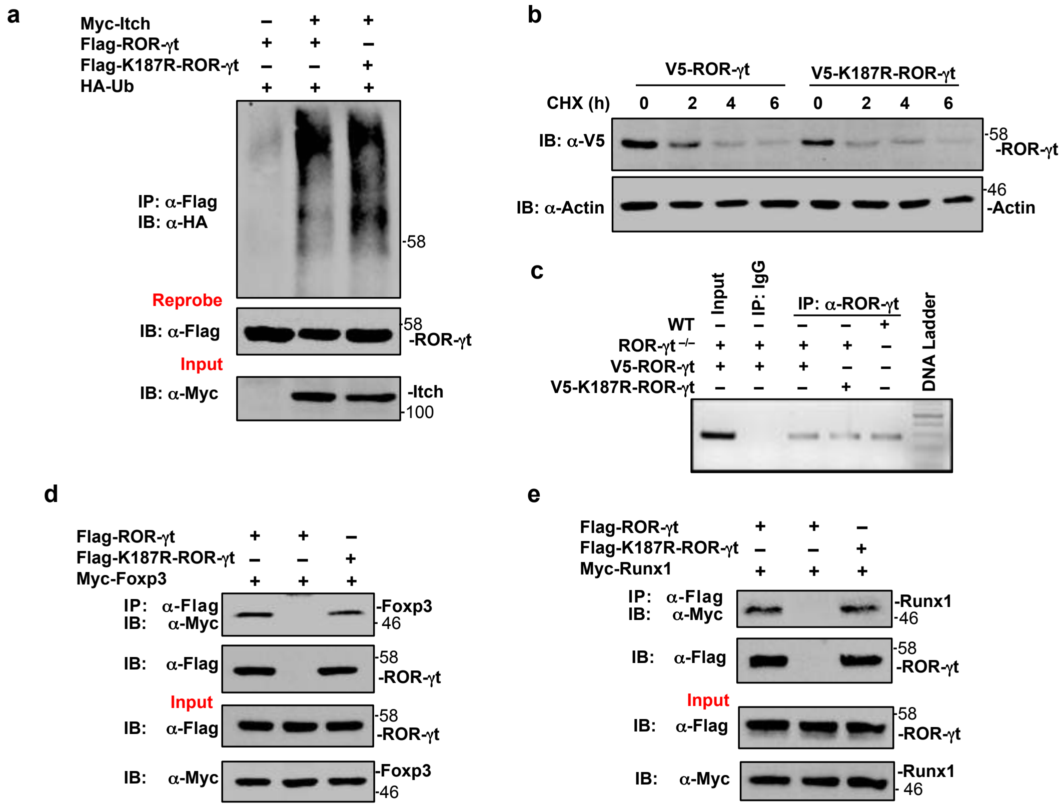

Supplementary Figure 5. SUMOylation of ROR-γt does not affect its ubiquitination, stability, DNA binding activity, and interaction with other binding partners. **(a)** Total cell lysates from 293T cells transfected with Myc-Itch, HA-Ub, and either Flag-ROR-γt or Flag-K187R-ROR-γt were immunoprecipitated with anti-Flag antibody. The immunoprecipitates were immunoblotted with anti-HA antibody to detect the ubiquitinated form of ROR-γt. **(b)** A cycloheximide chase experiment was performed in ROR-γt$^{-/-}$ cells transduced with lentivirus encoding V5-tagged WT-ROR-γt or K187R-ROR-γt. The expression of ROR-γt was analyzed by immunoblotting with anti-V5 antibody. **(c)** Binding of ROR-γt to the IL-17 promoter was analyzed by ChIP analysis using a DNA-protein complex from transduced CD4^{+} T cells expressing WT-ROR-γt or the SUMOylation-deficient mutant of ROR-γt (K187R-ROR-γt) and immunoprecipitated with anti–ROR-γt or control antibody (IgG). **(d-e)** Whole-cell lysates of 293T cells were transfected with plasmids encoding **(d)** Myc-Foxp3 or **(e)** Myc-Runx1 with either Flag-ROR-γt or Flag-K187R-ROR-γt. Lysates were immunoprecipitated with anti-Flag or the control IgG antibody and immunoblotted with anti-c-Myc antibody. The data are representative of three or more independent experiments.

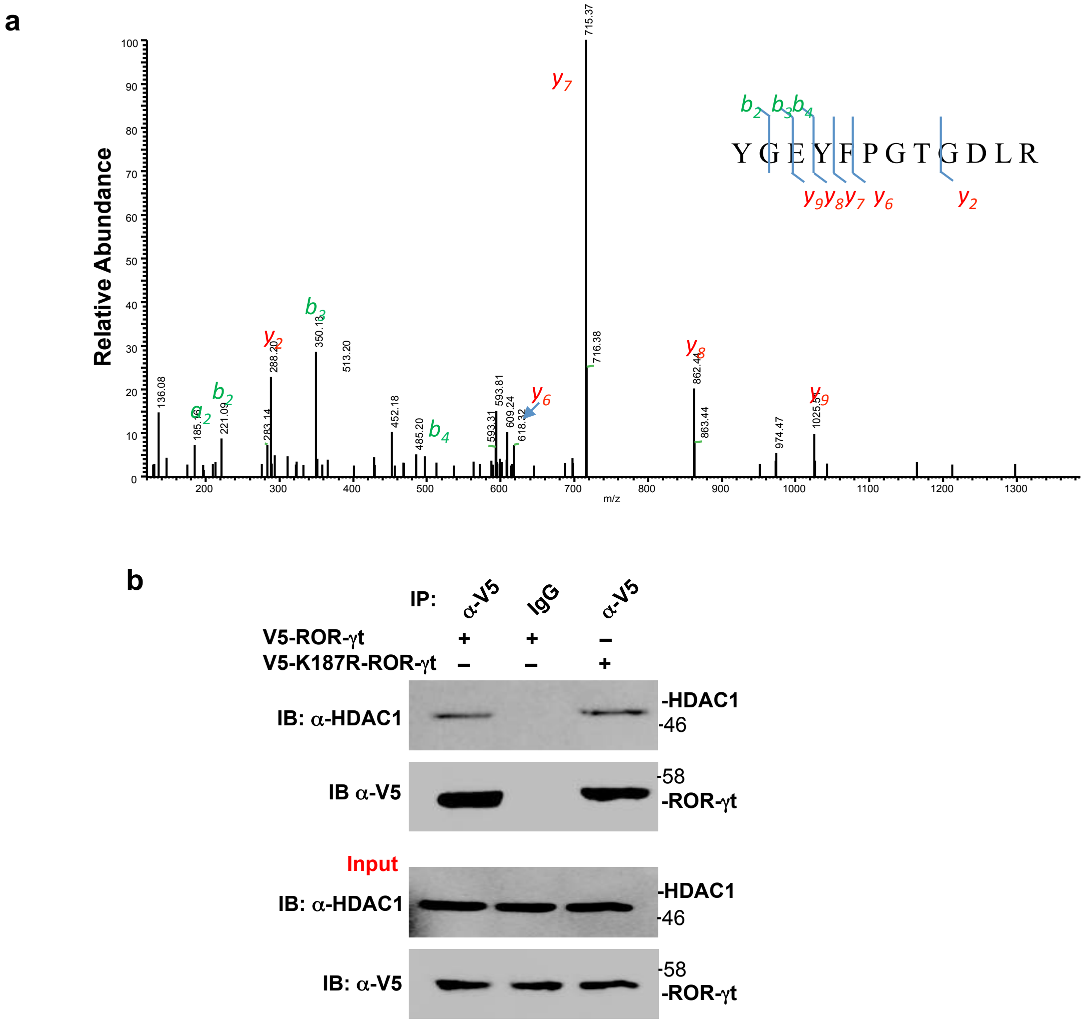

Supplementary Figure 6. HDAC2 interacts with ROR-γt. **(a)** Lysate was prepared from cLPLs of WT mice and subjected to immunoprecipitation with anti-ROR-γt antibody or control IgG antibody. The precipitated proteins were subjected to SDS-PAGE and in-gel digestion. The resulting peptides were analyzed by high-resolution MS/MS. HDAC2 (SwissProt #Q8BQ10) was identified as a specific interactor of ROR-γt protein. An MS/MS spectrum of the peptide ^{202}YGEYFPGTGDLR^{213} ($[M+H]^{+2}$ = 687.81 *m/z*) belonging to HDAC2 is shown. Observed *b*- and *y*-ions are indicated. **(b)** Interaction of WT and K187R-ROR-γt mutant with HDAC1. ROR-γt$^{-/-}$ CD4^{+} T cells were transduced with WT-ROR-γt or K187R-ROR-γt, immunoprecipitated with anti-V5 antibody, and immunoblotted with antibody against HDAC1. Data are representative of three or mroe independent experiments.

Gating strategies used for flow cytometry

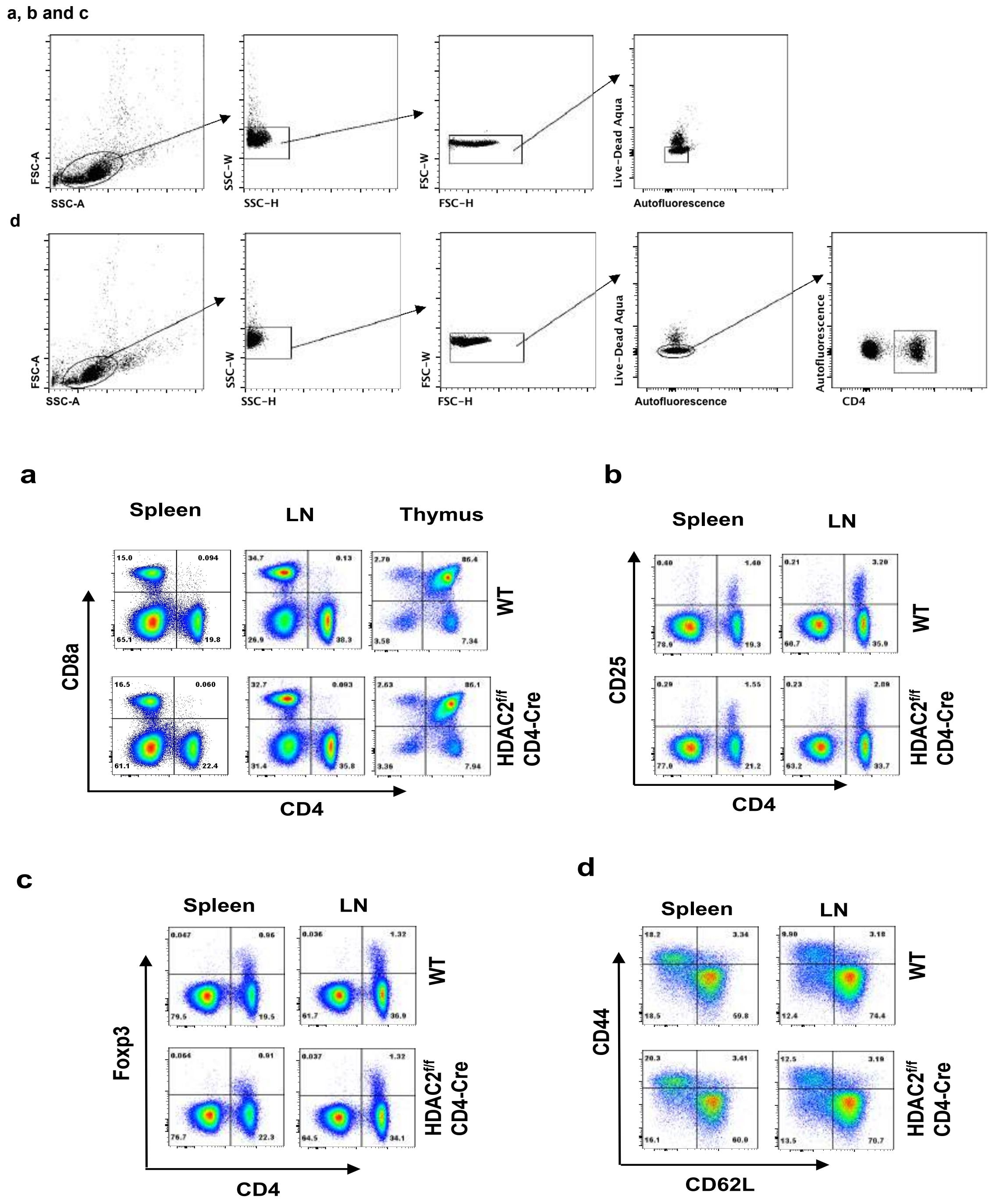

Supplementary Figure 7. Flow cytometric analysis of T cells in spleen, lymph node, and thymus of WT and HDAC2$^{f/f}$ CD4-Cre mice. **(a)** CD8a and CD4 plots from spleen, lymph node, and thymus are gated on live cells. **(b)** CD4 and CD25 plots from spleen and lymph node are gated on live cells. **(c)** CD4 and Foxp3 plots from spleen and lymph node are gated on live cells. **(d)** CD44 and CD62L plots are gated from CD4^{+} live cells. Data are representative of three independent experiments.

Gating strategies used for flow cytometry

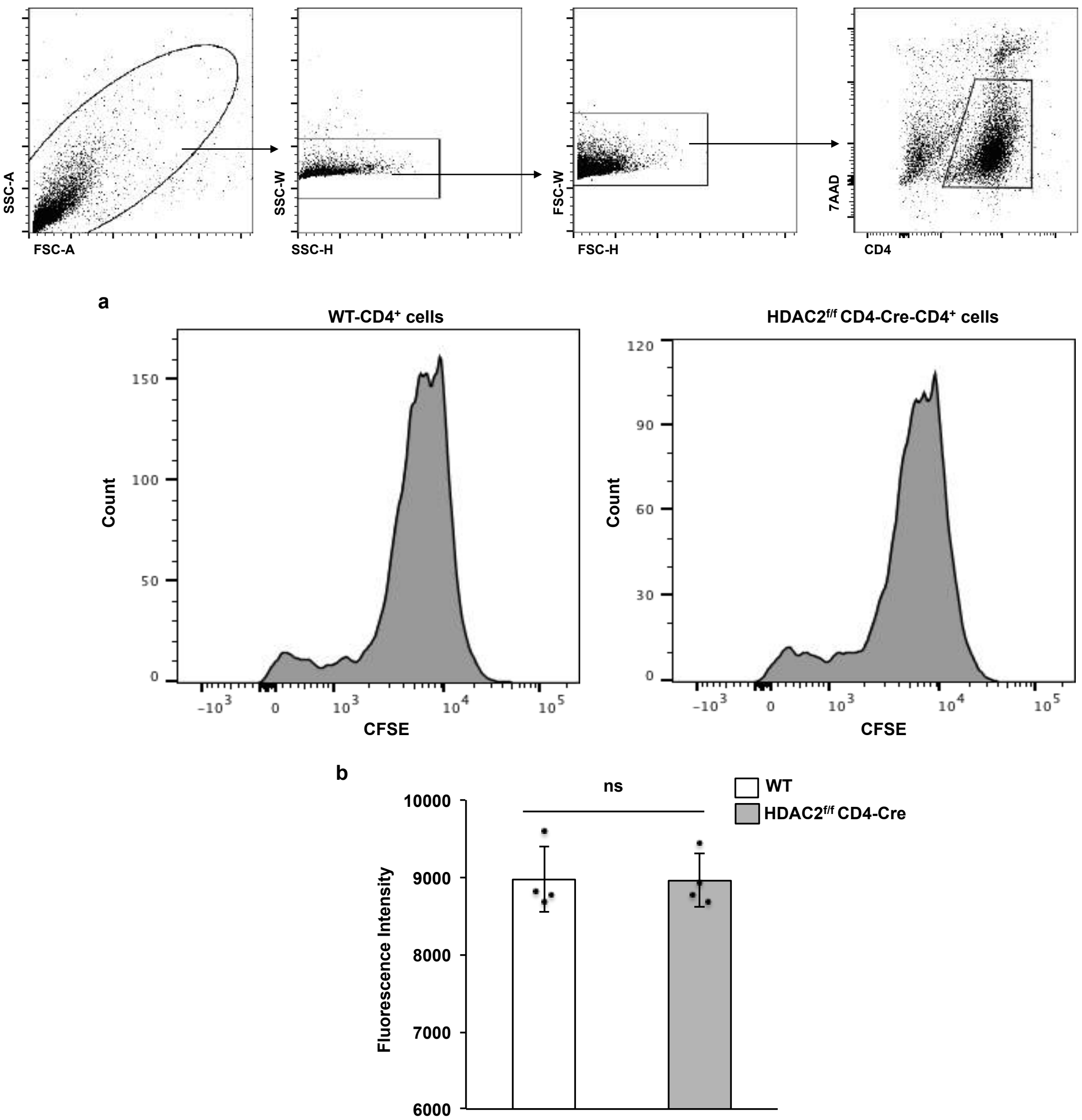

Supplementary Figure 8. Comparison of cell proliferation and cell viability of WT and $HDAC2^{-/-}$ $CD4^+$ T cells. (**a**) $CD4^+$ T cell proliferation was measured by CFSE dilution on day 3 after stimulation of $CD4^+$ T cells. **(b)** Cell viability was measured by using Resazurin Sodium Salt. Data are representative of three independent experiments. ($p > 0.05$) ns -non significant (two-tail *t* test) error bars are S.D.

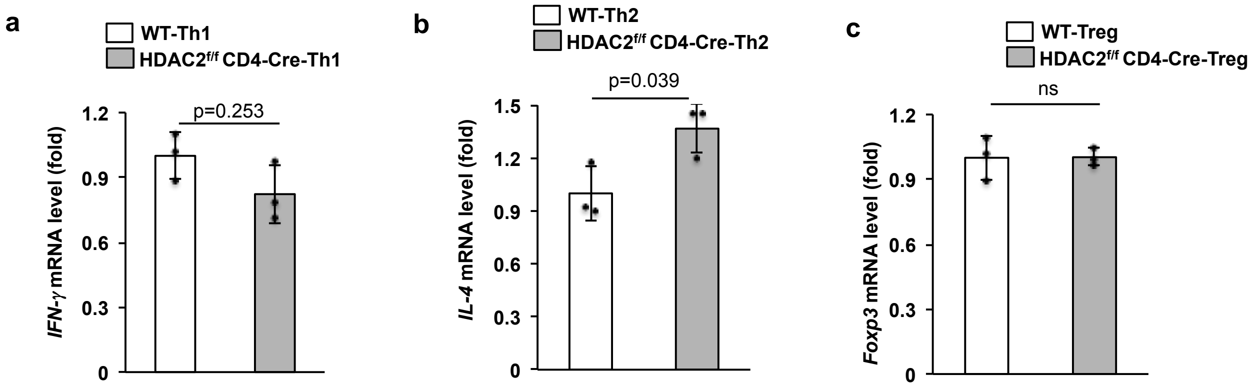

Supplementary Figure 9. Expression of **(a)** *IFN-γ*, **(b)** *IL-4,* and **(c)** *Foxp3* in WT and HDAC2$^{-/-}$ in *in vitro* generated Th1, Th2 and Tregs. CD4^{+} T cells from wild-type (WT) and HDAC2$^{f/f}$ CD4-Cre mice were differentiated under Th1, Th2 and Treg-polarizing conditions, and expression of *IFN-γ*, *IL-4,* and *Foxp3* was analyzed by real-time PCR. Data are representative of three independent experiments. ($p > 0.05$) ns -non significant (two-tail *t* test) error bars are S.D.

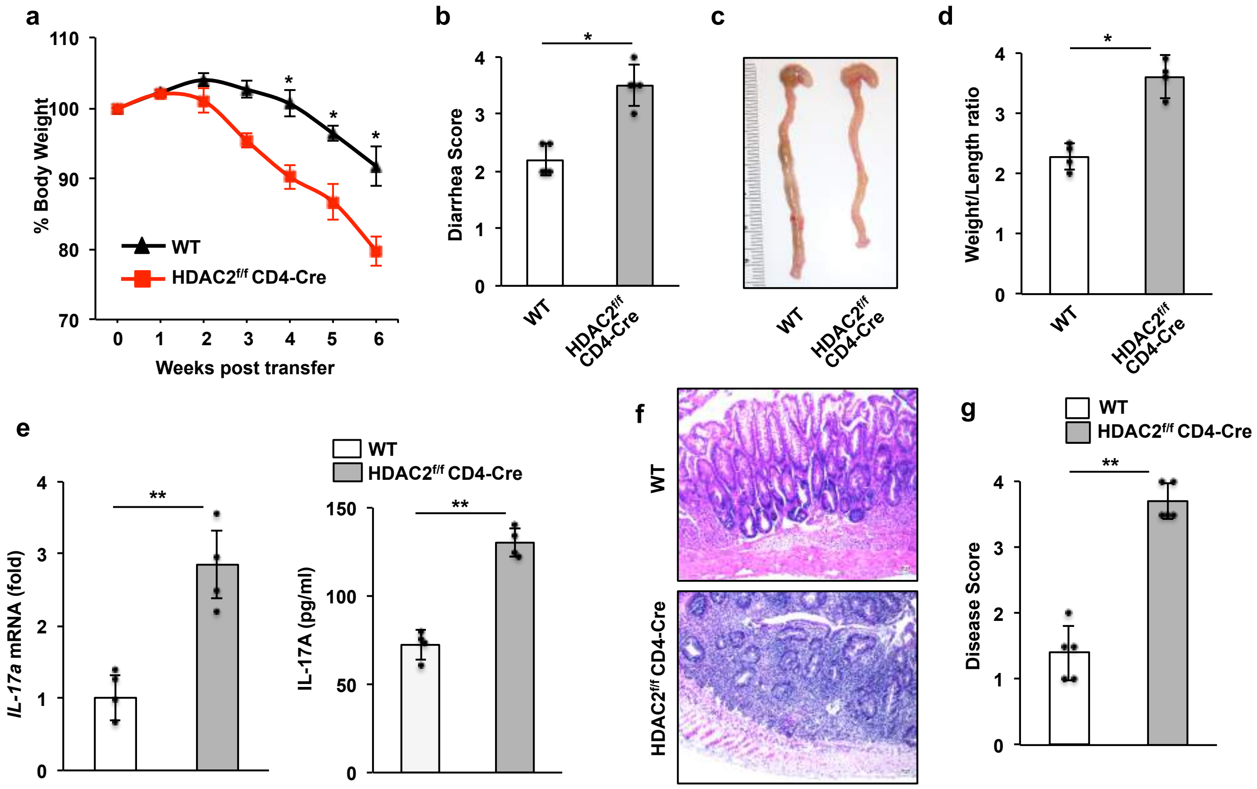

Supplementary Figure 10. HDAC2$^{f/f}$ CD4-Cre CD4^{+}CD45RB^{hi} cells induce enhanced colitis in Rag1$^{-/-}$ mice. Data show **(a)** body weight, (**b**) diarrhea score, (**c**) colon size, and (**d**) colonic weight-to-length ratio of Rag1$^{-/-}$ mice (n = 5 per group) given an intraperitoneal injection of CD4^{+}CD45RB^{hi} cells from WT or HDAC2$^{f/f}$ CD4-Cre mice. **(e)** Real-time PCR analysis was performed of *IL-17a* mRNA in the cLPLs, and ELISA was used to measure IL-17A secretion in the colon explant culture in Rag1$^{-/-}$ mice as in **a–c**. Results are presented relative to those of Rag1$^{-/-}$ mice given WT cells. **(f)** Microscopy is shown of H&E-stained colonic sections from Rag1$^{-/-}$ mice as in **a–c,** with **(g)** disease scores of those sections. Data are representative of three independent experiments. *p < 0.01, **p < 0.001 (two-tail t test) error bars are S.D.

.

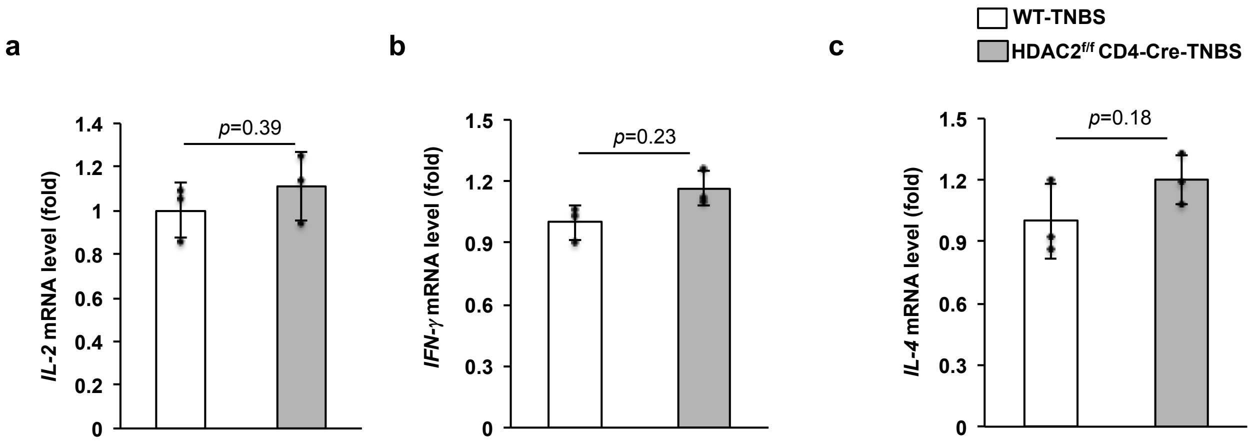

Supplementary Figure 11. Real-time PCR analysis for expression of **(a)** *IL-2*, **(b)** *IFN-γ*, and **(c)** *IL-4* in the colonic mucosa of WT and HDAC2$^{f/f}$ CD4-Cre mice treated with TNBS. Data are representative of three independent experiments. (p > 0.05) ns -non significant (two-tail t test) error bars are S.D.

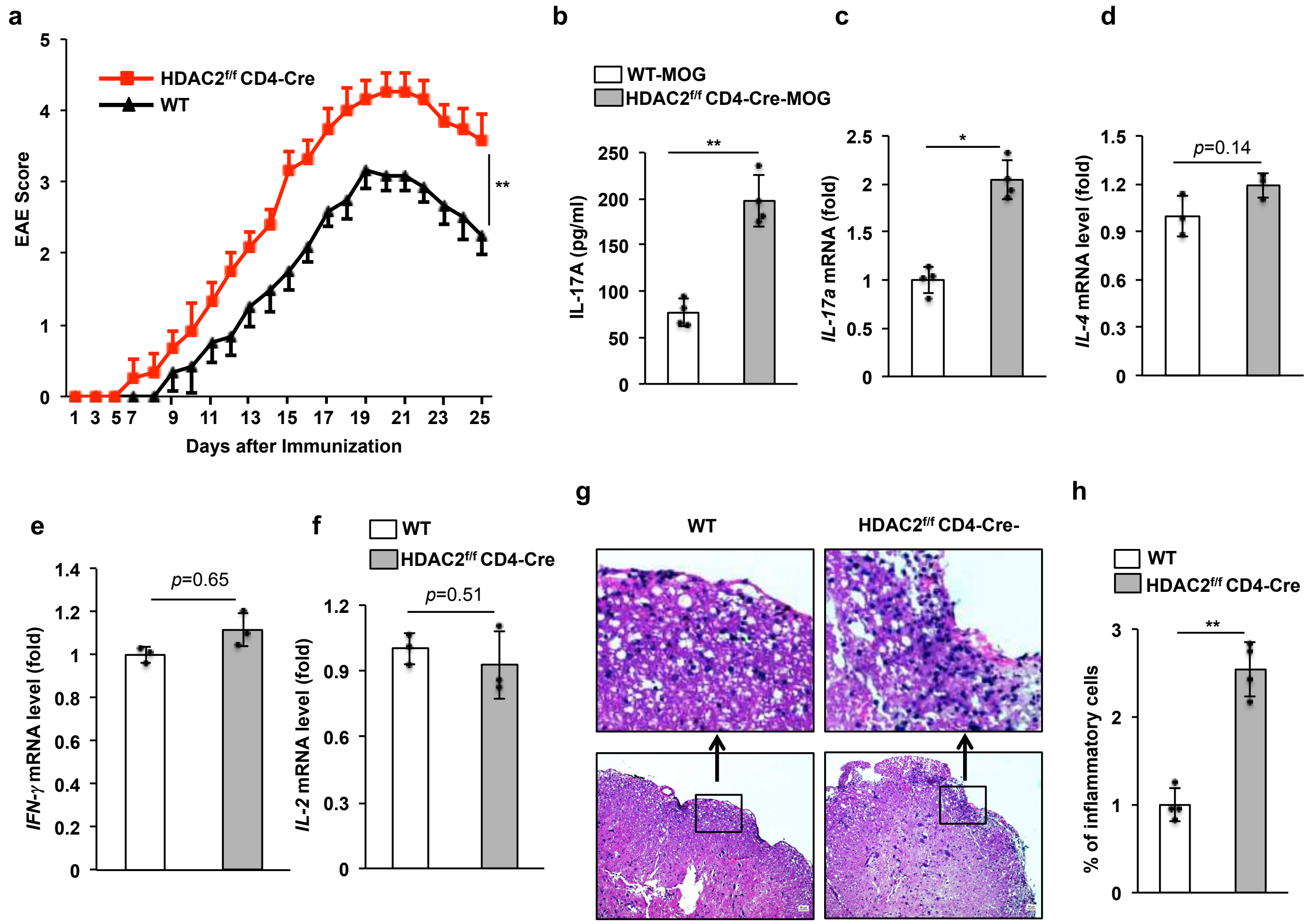

Supplementary Figure 12. WT and HDAC2$^{f/f}$ CD4-Cre (n = 5 per group) were immunized with MOG$_{35\text{-}55}$ peptide in CFA. Data show **(a)** clinical EAE scores (0-5-severity scale) and **(b)** ELISA to measure IL-17A secretion in splenocytes stimulated for 72 h with MOG$_{35\text{-}55}$ peptide. **(c-f)** Real-time PCR was performed to measure the mRNA level of *IL-17a*, *IL-4*, *IFN-γ* and *IL-2* in spinal cord. **(g)** H&E-stained sections of the spinal cord were prepared. **(h)** The percentage of inflammatory cell infiltration in the unit area was determined. Data are representative of three independent experiments. *$p < 0.01$, **$p < 0.001$ (two-tail t test) error bars are S.D.

Uncropped Blots

Figure. 1

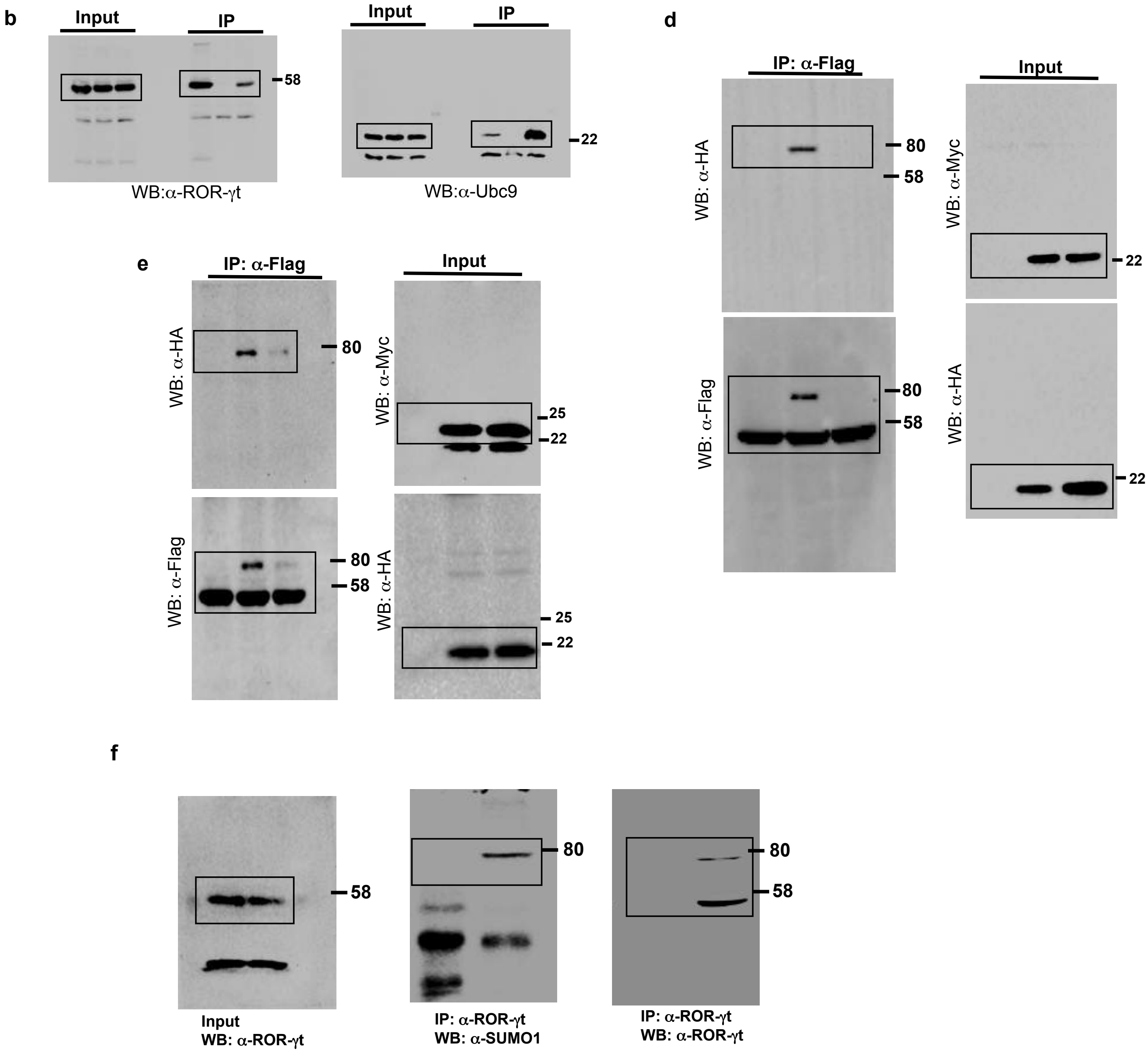

Figure. 2

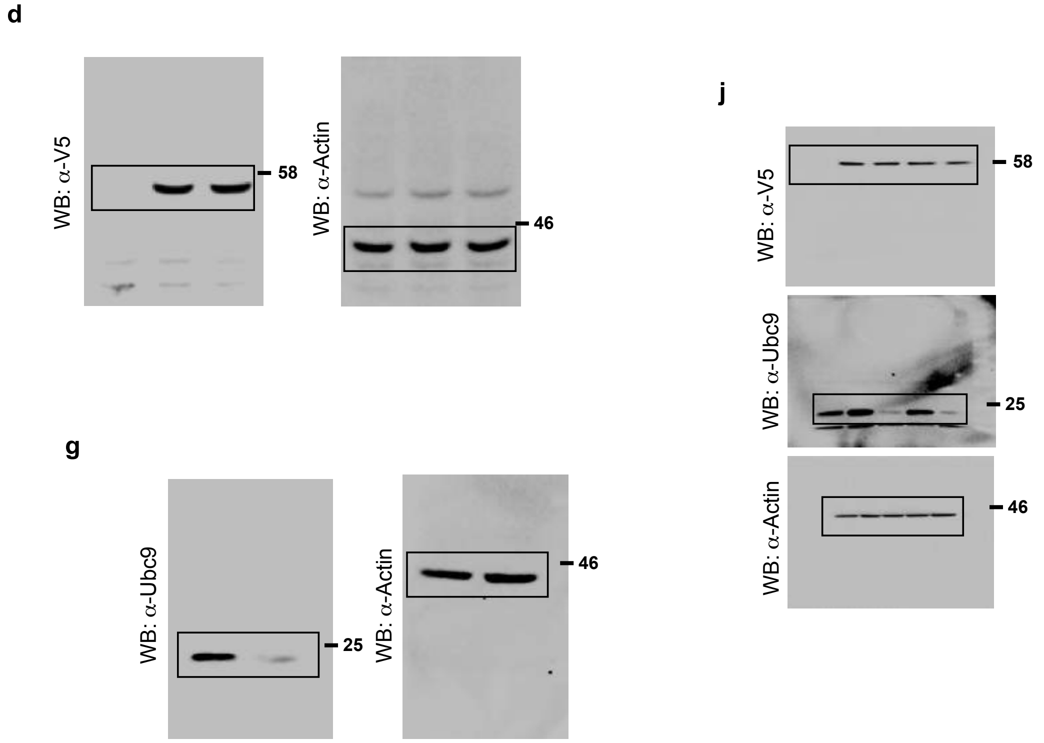

Figure. 4

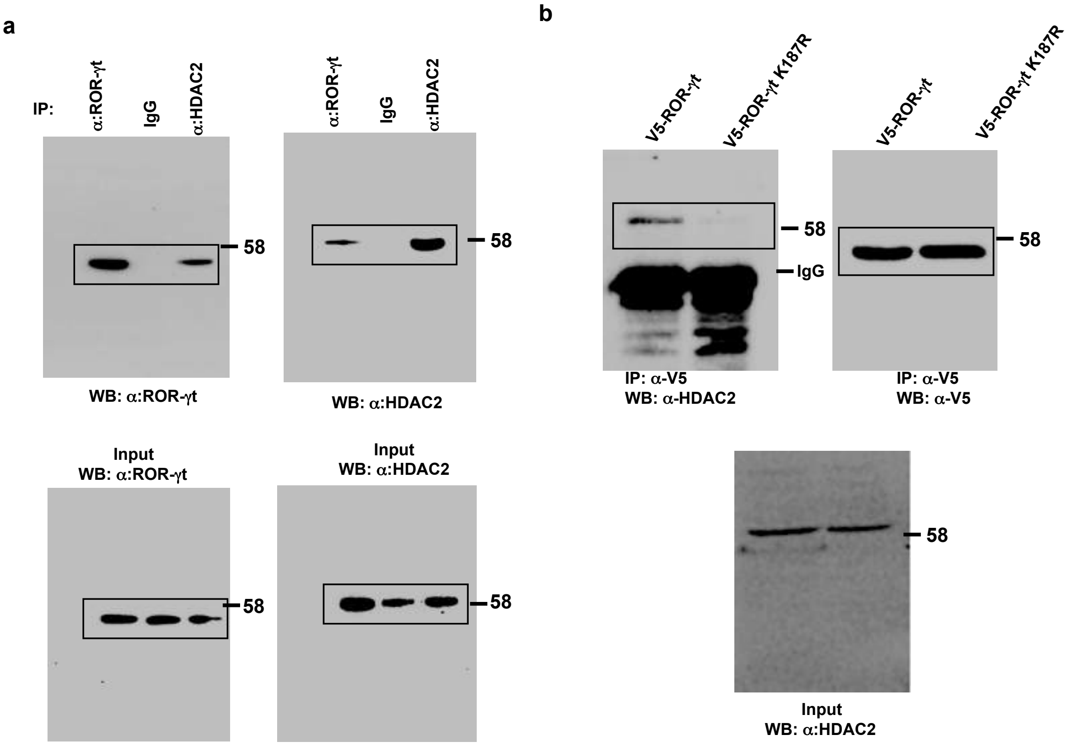

Figure. 5

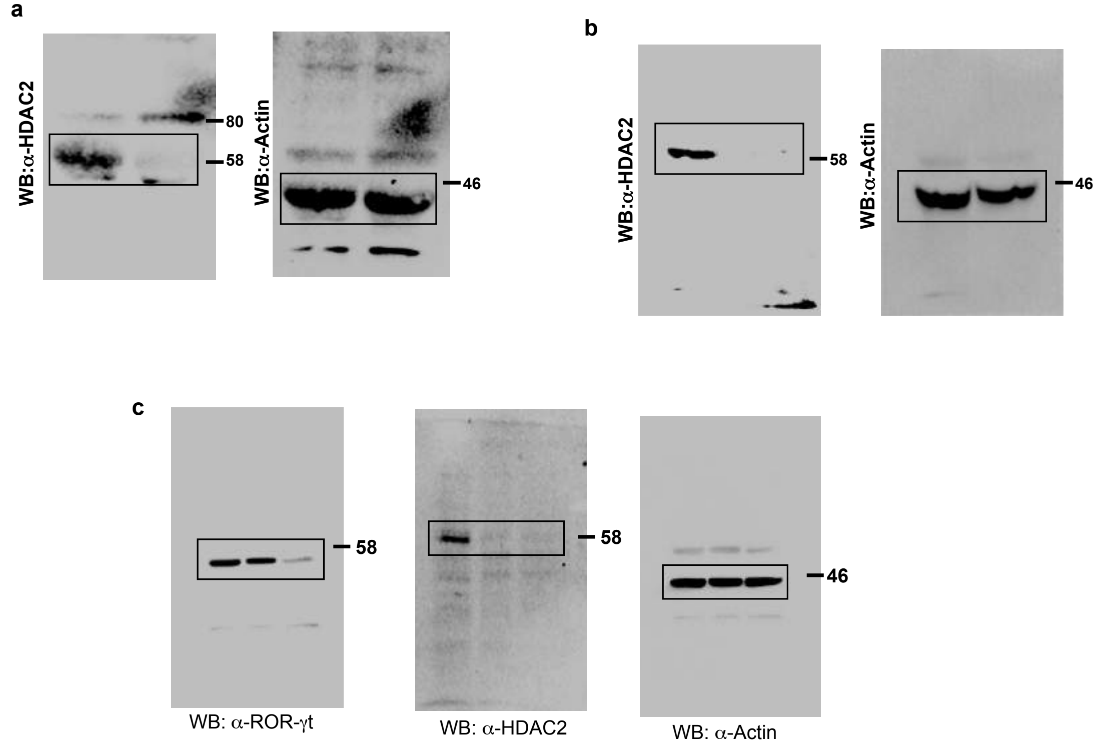

Supplementary Figure. 1

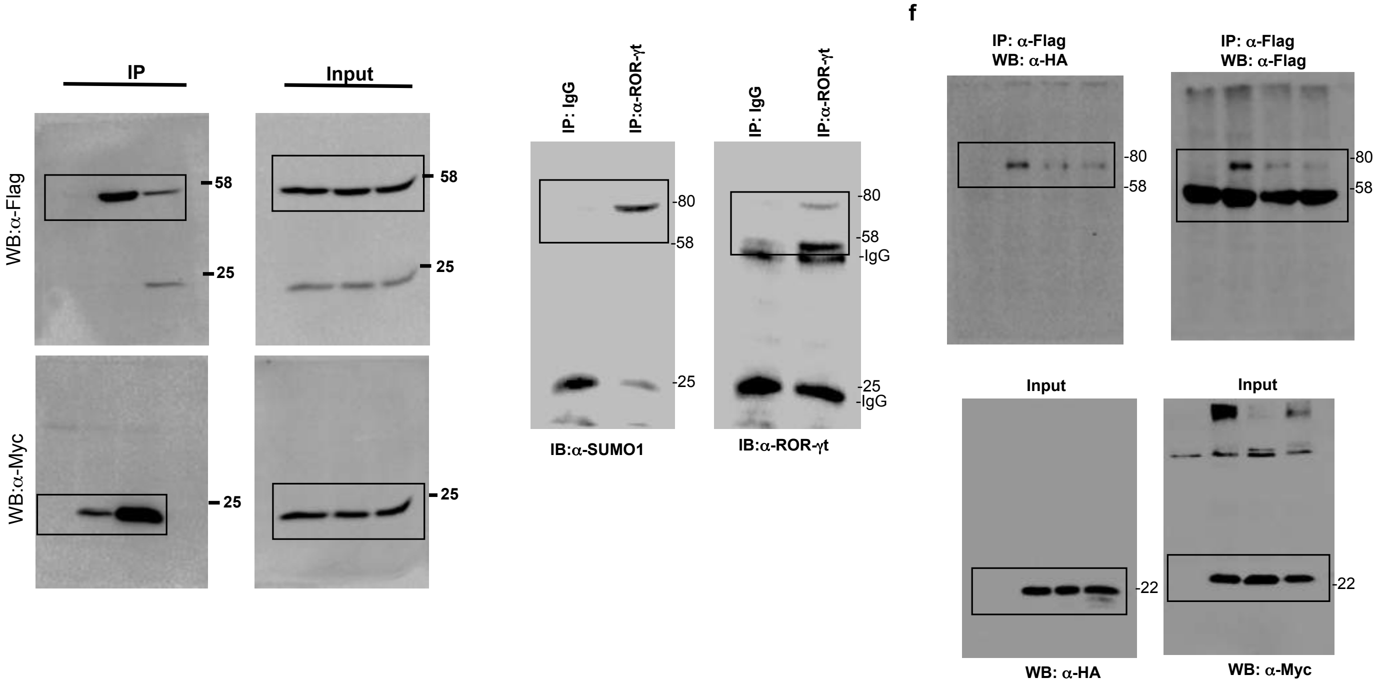

Supplementary Figure. 5

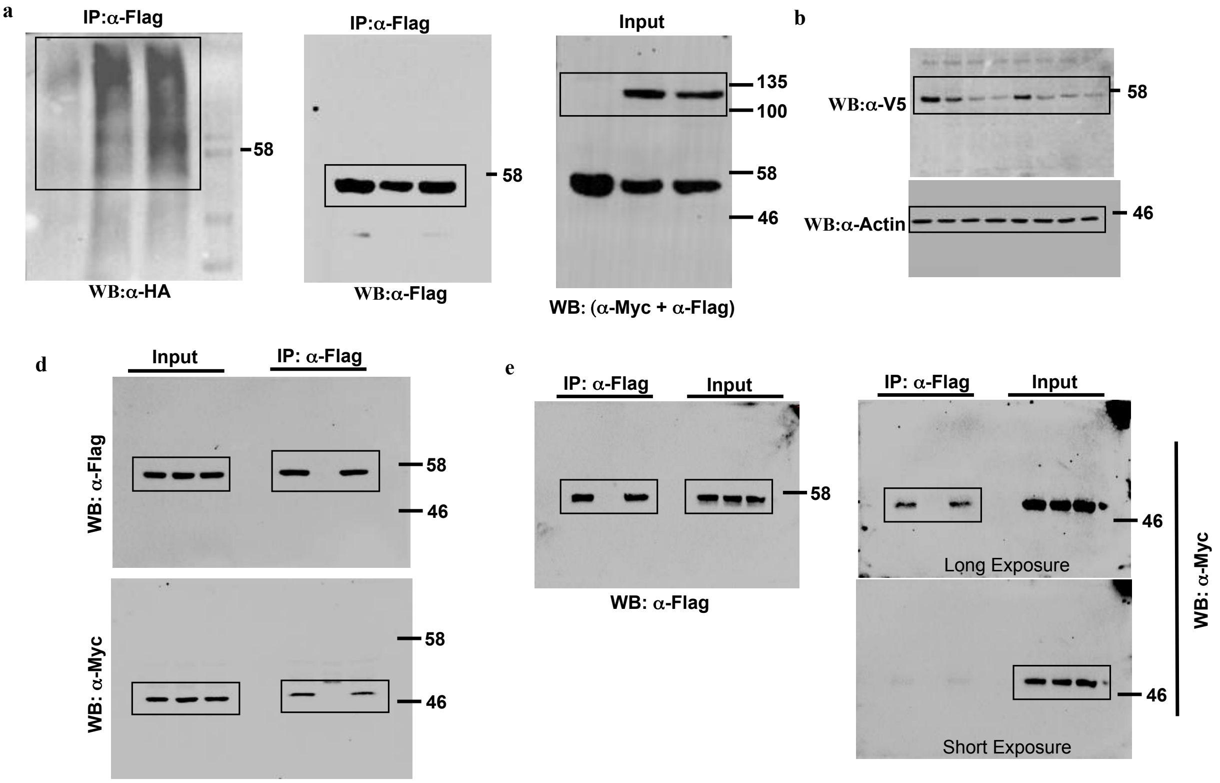

Supplementary Figure. 6

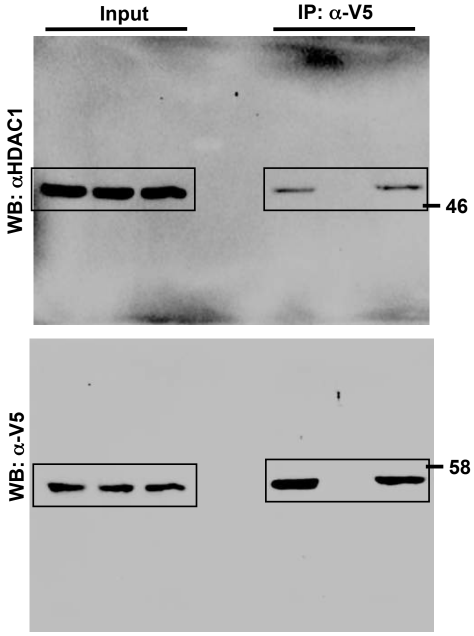

Supplement: Supplementary file 1 — Supplementary Information [file 41467_2018_6924_MOESM1_ESM.pdf]
